# Supplementary material for: Magnitude and associated factors of anti-retroviral therapy adherence among children attending HIV care and treatment clinics in Dar es Salaam, Tanzania
Source: PLoS One. 2022 Sep 30;17(9):e0275420. doi: 10.1371/journal.pone.0275420 (PMC9524636; doi:10.1371/journal.pone.0275420)
Supplement: S1 File — (DOCX) [file pone.0275420.s001.docx]

##

## S1 Appendix: The Questionnaire- English

**MAGNITUDE AND ASSOCIATED FACTORS OF ART ADHERENCE AMONG HIV-POSITIVE CHILDREN 1-14 YEARS ATTENDING HIV CARE AND TREATMENT CLINICS IN DAR ES SALAAM.**

**CRF number ______________________**

**Date of interview ___________________**

**Place of residence ___________________**

**Socio-demographic characteristics**

1. Sex of child a) Male b) Female
2. Age of child ____________
3. Age category of the child a)1-5 yrs b) 5-9 yrs c) 10-14 yrs
4. Schooling status of child a) Attending school b) Not attending school
5. If attending, what kind of school a) Day school b) Boarding School
6. Parental status a) both parents b) single parent c) non-parental caretaker

**Caregiver characteristics**

1. Age of caregiver__________________
2. Relation of caregiver a) Biological parent b) Non-biological parent
3. Care givers marital status a) Single b) Married c) Co-habiting d) Divorced/separated e) Widowed
4. Caregivers employment status a) Employed b) Unemployed
5. Average monthly income a) Less than 100,000 b) More than 100,000

**Anti-Retroviral Therapy use**

1. What ART is the child currently on?

a) ABC/3TC+ LPV/r ( ) b) AZT/3TC+LPV/r ( ) c) AZT/3TC/NVP ( )

d) AZT/3TC+EFV ( ) e) ABC/3TC+EFV ( ) f) TDF/3TC/EFV ( )

g) FTC/TDF+LPV/r ( )

1. How long has the child been on ARTs? a) Less than 2 yrs b) More than 2 yrs
2. How many pills does the child take per dose? a) 1 pill b) 2-3 pills c) more than 3 pills
3. How many times per day? a) once per day b) twice a day
4. Is the HIV status disclosed to the child? a) Yes b) No
5. Who is responsible for administering the drugs to the child? a) The child him/herself b) Primary caregiver only c) child and caregiver d) Others (specify)_______________

**Anti-Retroviral drug adherence**

1. Many people find it hard to administer the drug every single day. In the past 4 days how many days has the child missed taking all their doses

a) None b) One day c) Two days d) Three days e) Four days

1. On the scale of 1 to 10, mark a line on this scale of how the child took their pills over the past one month, 0 meaning none pills were taken, 5 means half doses were taken and 10 means each and every pill was taken over the whole month.


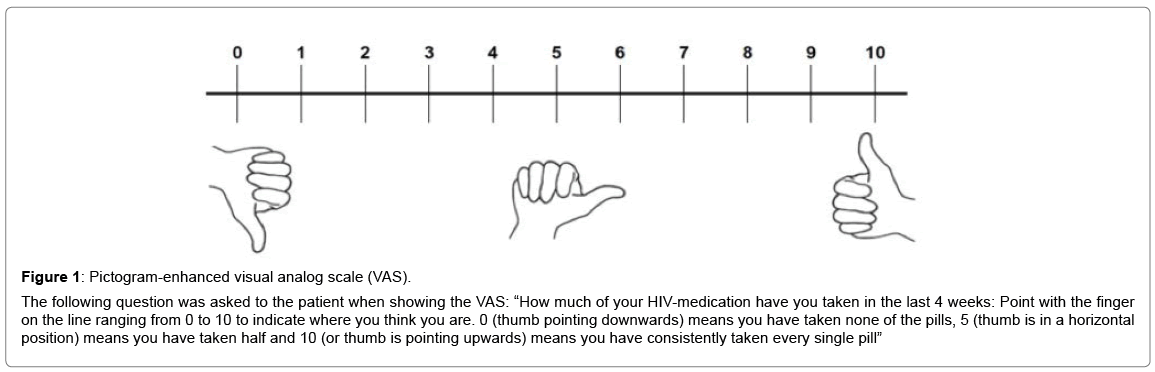


1. How many appointments did the child have scheduled over the past 6 months?

a) One b) Two c) three d) Four e) Five f) Six

1. How many did the child miss?
2. None b) One c)Two d) Three e) Four f) Five g) Six
3. Number of visits recorded in the patients card
4. One b) Two c) Three d) Four e) Five f) Six
5. Do you have the pill bottle with you? a) Yes b) No
6. If yes, No. of pills actually taken X 100 = ________%

No. of pills supposed to have been taken

**Factors affecting ART adherence**

1. Unwillingness to medicate in front of others a) Yes b) No
2. Side effects experienced a) Yes b) No
3. Childs inter-current illness a) Yes b) No
4. Availability of adequate food a) Yes b) No
5. Has your drug been changed due to unavailability? a) Yes b) No
6. Do you get adequate drug refill at each visit? a) Yes b) No
7. Do you get difficulty accessing the health Facility? a) Yes b) No
8. If yes, please specify ________________________________
9. Is the clinic timing convenient for you? a) Yes b) No
10. If no, please specify ________________________________
11. Have you been told about importance of adherence? a) Yes b) No
12. Do you believe in the effectiveness of ARTs? a) Yes b) No
13. Others (please specify) _________________________________________________

**Medical information (To be completed by the investigator)**

1. Height/length (cms) ____________
2. Weight (kgs)__________
3. MUAC (cms) _________ a) SAM b) MAM c) Normal
4. Weight/length (1-5 yrs)________a) SAM b) MAM c) Normal
5. BMI for age (>5 yrs)_________a) SAM b) MAM c) Normal
6. CD 4 count ________ a) Low b) High
7. Viral load _________ a) <1000 copies/ml b)≥1000 copies/ml
8. Clinical Stage at presentation a) stage 1 b) stage 2 c) stage 3 d) stage 4
9. Has the Child gotten any infection over the past six months? a) Yes b) No If yes please specify which one ____________________________

**THANKYOU FOR YOUR COOPERATION**

## The Questionnaire- Kiswahili

**MAGNITUDE AND ASSOCIATED FACTORS OF ART ADHERENCE AMONG HIV-POSITIVE CHILDREN 1-14 YEARS ATTENDING HIV CARE AND TREATMENT CLINICS IN DAR-ES-SALAAM**

Namba ya usajili ______________________

Kituo Cha Afya _______________________

Tarehe ya Dodoso ___________________

Makazi___________________

**Taarifa za muhusikazakijami**

1. Jinsia ya mtoto a) Kiume b) Kike
2. Umri wa mtoto ____________
3. Umri wa mtoto a) miaka 1-5 b) miaka 5-9 c) miaka 10-14
4. Hali ya shule ya mtoto a) Anaendashule b) Haendishule
5. Kama anaenda shule a) shule ya kutwa b) shule ya kulala
6. Hali ya wazazi a) wazazi wote wawili b) mzazi moja c) walezi

**Taarifa za mlezi**

1. Umri wa mlezi__________________
2. Usiano na mlezi a) mzazi b) mlezi
3. Hali ya ndoa a) ame(owa/olewa) b) haja(oa/olewa) c) wanaishipamoja d) wameachana e) mjane
4. Hali ya ajira a) ameajiriwa b) hajaajiriwa c) Amejiajiri
5. Kipato kwa mwezi a) Chini ya 100,000 b) Zaidi ya 100,000

**Matumizi ya dawa za VVU.**

1. Ni dawagani za VVU motto ana tumia?

a) ABC/3TC+ LPV/r ( ) b)AZT/3TC+LPV/r ( ) c) AZT/3TC/NVP ( )

d) AZT/3TC+EFV ( ) e) ABC/3TC+EFV ( ) f) TDF/3TC/EFV ( )

g) FTC/TDF+LPV/r ( )

1. Ana tumia dawa hizi kwamuda gani? a) chini ya miaka 2 b) zaidi ya miaka miwili
2. Ni vidonge vingapi ambavyo mtoto hu anachukuwa kwa dozi? a) kidonge 1 b) vidonge 2-3 c) zaidi ya vidonge 3
3. Mara ngapi kwasiku a) mara moja b) mara mbhili
4. Je, hali ya VVU inajulikana kwa mtoto? a) ndio b) hapana
5. Nani anampa mtoto dawa? a) mtoto mwenyewe b) mlezi mwenyewe c) mtoto na mlezi d) wengine (taja)_______________

**Uzingatiajiwadawa za VVU.**

1. Watu wengi wanaona ugumu ku tumia dawa kilasiku, Je ndani ya siku 4 zilizopita mtoto ame kosa dawa kwasiku ngapi?

a) haja kosa kabisa b) siku 1 c) siku 2 d) siku 3 e) siku 4

1. Kwa kiwango cha 1-10, weka alama ya kiwango ambacho mtoto ametumia dawa za VVU ndani ya mwezi moja uliopita, 0 inamaanisha hakuna dawa zilizo chukuliwa, 5 inamaanisha nusu ya dozi ilichukuli wa na 10 inamaanisha kila kidonge kilichukuliwa.


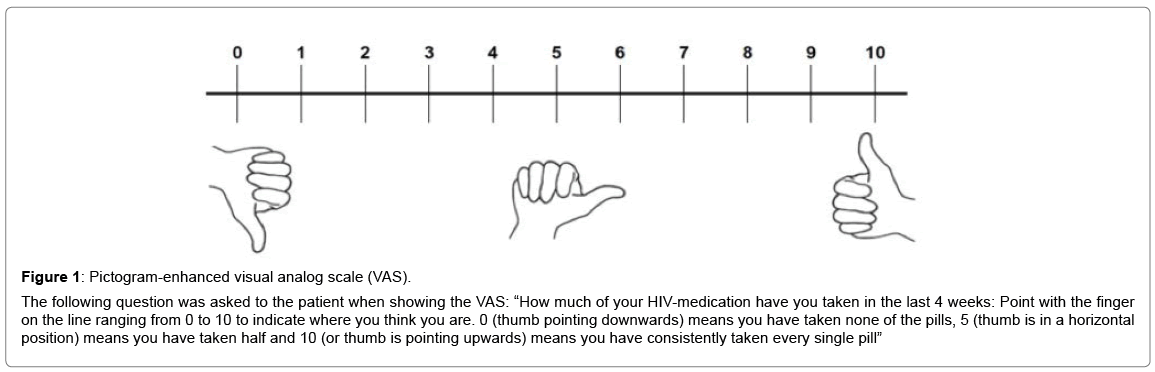


1. Mtoto alipangi wakuhudhuria kliniki mara ngapi ndani ya miezi 6 iliyopita?

a) 1 b) 2 c) 3 d) 4 e) 5 f) 6

1. Alikosa mara ngapi?
2. 0 b) 1 c) 2 d) 3 e) 4 f) 5 g) 6
3. Hidadi ya maudhurio ilio recodiwa na duka la dawa?

a) 1 b) 2 c) 3 d) 4 e) 5 f) 6

1. Je, unacho kikopo chako cha vidonge? a) Ndio b) hapana
2. Kama ndio, idadi ya vidongevilivyotumika X 100 = ________ %

Idadi ya vidongevinavyotakiwakutumika

**Mambo yanayo athiri uzingatiaji wa dawa za VVU**

1. Kuto kuwa tayari ku tumia dawa mbele ya watu a) ndio b) hapana
2. Madhara ya dawa a) ndio b) hapana
3. Mtoto ku umwa a) ndio b) hapana
4. Upatikaniaji wachakula nyumbani a) ndio b) hapana
5. Dawa kubadlishwa kutokana nakuto patikana a) ndio b) hapana
6. Unapata dawa za kutosha kila unapokuja kuongeza dawa a) ndio b) hapana
7. Je, unapata shida kufika kliniki a) ndio b) hapana
8. Kama ndio taja shida gani__________________________
9. Muda ya kliniki unakufaa? a) ndio b) hapana
10. Kama Hapana taja mapendekezo yako ________________________
11. Umeambiwa umuhimu kuzingatia dawa za VVU a) ndio b) hapana
12. Je, unaamini hizo dawa zinam saidia mototo a) ndio b) hapana c) sijui
13. Mengine (taja) _________________________________________________

**Medical information (To be completed by the investigator)**

1. Height/length (cms) ____________
2. Weight (kgs)__________
3. MUAC (cms) _________ a) SAM b) MAM c) Normal
4. Weight/length (1-5yrs)________ a) SAM b) MAM c) Normal
5. BMI for age (>5 yrs)_________ a) SAM b) MAM c) Normal
6. CD 4 count ________ a) Low b) High
7. Viral load _________ a) <1000 copies/ml b)≥1000 copies/ml
8. Clinical Stage at presentation a) stage 1 b) stage 2 c) stage 3 d) stage 4
9. Has the Child gotten any infection over the past six months? a) Yes b) No If yes please specify which one ____________________________

**ASANTE KWA USHIRIKIANO**
