# Supplementary material for: Magnitude and associated factors of anti-retroviral therapy adherence among children attending HIV care and treatment clinics in Dar es Salaam, Tanzania
Source: PLoS One. 2022 Sep 30;17(9):e0275420. doi: 10.1371/journal.pone.0275420 (PMC9524636; doi:10.1371/journal.pone.0275420)
Supplement: Attachment — Submitted filename: PONE-D-20-25583_reviewer.pdf [file pone.0275420.s003.pdf]

PLOS ONE

MAGNITUDE AND ATTRIBUTES OF ANTI-RETROVIRAL THERAPY ADHERENCE AMONG CHILDREN (1-14 YEARS) ATTENDING HIV CARE AND TREATMENT CLINICS IN DAR ES SALAAM, TANZANIA

--Manuscript Draft--

**Manuscript Number:**	PONE-D-20-25583
**Article Type:**	Research Article
**Full Title:**	MAGNITUDE AND ATTRIBUTES OF ANTI-RETROVIRAL THERAPY ADHERENCE AMONG CHILDREN (1-14 YEARS) ATTENDING HIV CARE AND TREATMENT CLINICS IN DAR ES SALAAM, TANZANIA
**Short Title:**	MAGNITUDE AND ATTRIBUTES OF ANTI-RETROVIRAL THERAPY ADHERENCE AMONG CHILDREN (1-14 YEARS) WITH HIV/AIDS
**Corresponding Author:**	Fatima Mehdi Mussa, MD, Mmed<br>Muhimbili University of Health and Allied Sciences<br>Dar es Salaam, TANZANIA, UNITED REPUBLIC OF
**Keywords:**	ART adherence among children; overall good adherence (≥95%); Four day self-report; one month self-recall report; missed clinic appointments; magnitude of adherence; factors associated with poor adherence
**Abstract:**	Introduction- The HIV epidemic continues to contribute significantly towards childhood mortality and morbidity. The up-scaling of the Anti-retroviral therapy (ART) access has seen more children surviving. In order to achieve the 90-90-90 goal of sustainable viral suppression, great effort needs to be made on ensuring adherence.<br>Objectives- To determine the adherence magnitude and associated factors of HIV positive children (1-14 years) attending HIV care and treatment clinics in Dar es Salaam.<br>Methods- A cross sectional study in HIV care and treatment clinics in Dar es Salaam. HIV infected children aged 1-14 years who had been on treatment for at least six months were consecutively enrolled. Four day self-report, one month self-recall report and missed clinic appointments were used to assess adherence.<br>Results- 333 participants were recruited. The overall good adherence (≥95%) was approximated to be 60% when subjected to all three measures. On multivariate logistic regression, factors associated with poor adherence were found to be caregivers aged less than 25 years [OR- 3.520, 95%CI- (1.471-8.422), p=0.005], children having an inter-current illness [OR- 10.869, 95% CI-(2.340-50.489), p=0.002] while, belief in ART effectiveness [OR-0.182, 95%CI-(0.055-0.599), p=0.005] and less advanced clinical stages (1 and 2)[OR- 0.507, 95%CI-(0.287-0.813) p=0.019] were less likely to be associated with poor adherence. The major reasons reported by caregivers for missing medications included forgetfulness (41%), high pill burden (21%), busy schedule (11%) and long waiting hours at clinic (9%).<br>Conclusion and recommendations- In the urban setting of Dar es Salaam, ART adherence was found to be relatively low using multiple adherence measures. Healthcare workers should assess and emphasise on ART adherence as an ongoing process and elicit barriers to medication administration. Strategies to maximize adherence should be targeted towards educating caregivers and providing additional support to special groups including younger caregivers, children with concomitant illness and advanced HIV clinical stage.
**Order of Authors:**	Fatima Mehdi Mussa, MD, Mmed
	HIGGINS P. MASSAWE
	HUSSEIN BHALLOO
	SIBTAIN MOLEDINA
	EVELYNE N. ASSENGA
**Additional Information:**	
**Question**	**Response**

Powered by Editorial Manager® and ProduXion Manager® from Aries Systems Corporation

**Financial Disclosure**<br><br>Enter a financial disclosure statement that describes the sources of funding for the work included in this submission. Review the submission guidelines for detailed requirements. View published research articles from *PLOS ONE* for specific examples.<br><br>This statement is required for submission and **will appear in the published article** if the submission is accepted. Please make sure it is accurate.<br><br>**Unfunded studies**<br>Enter: *The author(s) received no specific funding for this work.*<br><br>**Funded studies**<br>Enter a statement with the following details:<br>• Initials of the authors who received each award<br>• Grant numbers awarded to each author<br>• The full name of each funder<br>• URL of each funder website<br>• Did the sponsors or funders play any role in the study design, data collection and analysis, decision to publish, or preparation of the manuscript?<br>• **NO** - Include this sentence at the end of your statement: *The funders had no role in study design, data collection and analysis, decision to publish, or preparation of the manuscript.*<br>• **YES** - Specify the role(s) played.<br><br>* typeset	The funders had no role in study design, data collection and analysis, decision to publish, or preparation of the manuscript.
**Competing Interests**<br><br>Use the instructions below to enter a competing interest statement for this submission. On behalf of all authors, disclose any competing interests that could be perceived to bias this work—acknowledging all financial support and any other relevant financial or non-financial competing interests.<br><br>This statement **will appear in the published article** if the submission is accepted. Please make sure it is	The authors have declared that no competing interests exist

accurate. View published research articles from *PLOS ONE* for specific examples.<br><br>**NO authors have competing interests**<br><br>Enter: *The authors have declared that no competing interests exist.*<br><br>**Authors with competing interests**<br><br>Enter competing interest details beginning with this statement:<br><br>*I have read the journal's policy and the authors of this manuscript have the following competing interests: [insert competing interests here]*<br><br>* typeset	
**Ethics Statement**<br><br>Enter an ethics statement for this submission. This statement is required if the study involved:<br><br>• Human participants<br>• Human specimens or tissue<br>• Vertebrate animals or cephalopods<br>• Vertebrate embryos or tissues<br>• Field research<br><br>Write "N/A" if the submission does not require an ethics statement.<br><br>General guidance is provided below. Consult the submission guidelines for detailed instructions. **Make sure that all information entered here is included in the Methods section of the manuscript.**	Muhimbili University Of Health and Allied Sciences ethical review board, Ref No. D.A.287/298/01A/ Approval of ethical clearance for the study.<br>A written consent was obtained from the parent/guardian of the participants prior to recruitment into the study and for children 7 years and above with the capacity to understand, a formal written assent was further sought.

Powered by Editorial Manager® and ProduXion Manager® from Aries Systems Corporation

**Format for specific study types**<br><br>**Human Subject Research (involving human participants and/or tissue)**<br>• Give the name of the institutional review board or ethics committee that approved the study<br>• Include the approval number and/or a statement indicating approval of this research<br>• Indicate the form of consent obtained (written/oral) or the reason that consent was not obtained (e.g. the data were analyzed anonymously)<br><br>**Animal Research (involving vertebrate animals, embryos or tissues)**<br>• Provide the name of the Institutional Animal Care and Use Committee (IACUC) or other relevant ethics board that reviewed the study protocol, and indicate whether they approved this research or granted a formal waiver of ethical approval<br>• Include an approval number if one was obtained<br>• If the study involved *non-human primates*, add *additional details* about animal welfare and steps taken to ameliorate suffering<br>• If anesthesia, euthanasia, or any kind of animal sacrifice is part of the study, include briefly which substances and/or methods were applied<br><br>**Field Research**<br><br>Include the following details if this study involves the collection of plant, animal, or other materials from a natural setting:<br>• Field permit number<br>• Name of the institution or relevant body that granted permission	
**Data Availability**<br><br>Authors are required to make all data underlying the findings described fully available, without restriction, and from the time of publication. PLOS allows rare exceptions to address legal and ethical concerns. See the PLOS Data Policy and FAQ for detailed information.	No - some restrictions will apply

A Data Availability Statement describing where the data can be found is required at submission. Your answers to this question constitute the Data Availability Statement and **will be published in the article**, if accepted.<br><br>**Important:** Stating 'data available on request from the author' is not sufficient. If your data are only available upon request, select 'No' for the first question and explain your exceptional situation in the text box.<br><br>Do the authors confirm that all data underlying the findings described in their manuscript are fully available without restriction?	
**Describe where the data may be found in full sentences. If you are copying our sample text, replace any instances of XXX with the appropriate details.**<br><br>• If the data are **held or will be held in a public repository**, include URLs, accession numbers or DOIs. If this information will only be available after acceptance, indicate this by ticking the box below. For example: *All XXX files are available from the XXX database (accession number(s) XXX, XXX.).*<br>• If the data are all contained **within the manuscript and/or Supporting Information files**, enter the following: *All relevant data are within the manuscript and its Supporting Information files.*<br>• If neither of these applies but you are able to provide **details of access elsewhere**, with or without limitations, please do so. For example:<br><br>*Data cannot be shared publicly because of [XXX]. Data are available from the XXX Institutional Data Access / Ethics Committee (contact via XXX) for researchers who meet the criteria for access to confidential data.*<br><br>*The data underlying the results presented in the study are available from (include the name of the third party*	Data cannot be shared publicly because of confidentiality issues in HIV care and treatment. However, the data is safely stored and will be available for researchers who meet the criteria for access to confidential data.

and contact information or URL).<br>• This text is appropriate if the data are owned by a third party and authors do not have permission to share the data.<br><br>* typeset	
Additional data availability information:	Tick here if your circumstances are not covered by the questions above and you need the journal's help to make your data available.

Manuscript Click here to access/download;Manuscript;Manuscript PDF.pdf

MAGNITUDE AND ATTRIBUTES OF ANTI-RETROVIRAL THRAPY ADHERENCE AMONG CHILDREN ATTENDING HIV CARE AND TREATMENT CLINICS IN DAR ES SALAAM, TANZANIA

FATIMA M. MUSSA[1], HIGGINS P. MASSAWE[2], HUSSEIN BHALLOO[3], SIBTAIN MOLEDINA[4], EVELYNE N. ASSENGA[1]

CORRESPONDANCE SHOULD BE ADRESSED TO FATIMA M. MUSSA, f_mussa@icloud.com

1.Department of Paediatrics and Child Health, Muhimbili University of Health Sciences, Dar es Salaam, Tanzania

2. Department of Paediatrics and Child Health, Muhimbili National Hospital, Dar es Salaam, Tanzania

3. Sanitas Hospital, Dar es Salaam, Tanzania

4. Shree Hindu Mandal Hospital, Dar es Salaam, Tanzania

ABSTRACT

Introduction-The HIV epidemic continues to contribute significantly towards childhood mortality and morbidity. The up-scaling of the Anti-retroviral therapy (ART) access has seen more children surviving. In order to achieve the 90-90-90 goal of sustainable viral suppression, great effort needs to be made on ensuring adherence.

Objectives-To determine the adherence magnitude and associated factors of HIV positive children (1-14 years) attending HIV care and treatment clinics in Dar es Salaam.

Methods-A cross sectional study in HIV care and treatment clinics in Dar es Salaam. HIV infected children aged 1-14 years who had been on treatment for at least six months were consecutively enrolled. Four day self-report, one month self-recall report and missed clinic appointments were used to assess adherence.

Results-333 participants were recruited. The overall good adherence (≥95%) was approximated to be 60% when subjected to all three measures. On multivariate logistic regression, factors associated with poor adherence were found to be caregivers aged less than 25 years [OR- 3.520, 95%CI- (1.471-8.422), p=0.005], children having an inter-current illness [OR- 10.869, 95% CI-(2.340-50.489), p=0.002] while, belief in ART effectiveness [OR-0.182, 95%CI-(0.055-0.599), p=0.005] and less advanced clinical stages (1 and 2)[OR-0.507, 95%CI-(0.287-0.813) p=0.019] were less likely to be associated with poor adherence. The major reasons reported by caregivers for missing medications included forgetfulness (41%), high pill burden (21%), busy schedule (11%) and long waiting hours at clinic (9%).

Conclusion and recommendations- In the urban setting of Dar es Salaam, ART adherence was found to be relatively low using multiple adherence measures. Healthcare workers should assess and emphasise on ART adherence as an ongoing process and elicit barriers to medication administration. Strategies to maximize adherence should be targeted towards educating caregivers and providing additional support to special groups including younger caregivers, children with concomitant illness and advanced HIV clinical stage.

INTRODUCTION

The HIV epidemic continues to have a devastating consequence globally. According to UNAIDS, in 2018, 37.9 million people were living with HIV/AIDS worldwide, of which 1.7 million (5% of the population) were children aged less than 15 years(1). Sub-Saharan Africa bears the largest burden of the HIV epidemic, accounting for 1.9 million children living with HIV/AIDS and more than 60% of new infections occur in women, infants or young children(2). With the recent scale up of ART access in sub-Saharan countries, maximum focus needs to be laid on maintaining optimal adherence as it has been proven that, for greatest success in ART effectiveness(3), adherence should be at 95% or greater(4–6). The need for near-perfect adherence to a lifelong therapy from an early age has been identified as a major challenge in the administration of ART to HIV infected children(4,7,8). Poor adherence leads to low therapeutic drug levels, resulting in incomplete suppression of HIV replication. It leads to resistance to the drugs and moreover cross resistance within the same class(5,6), thus compromising future treatment options and increasing mortality. Adherence reports among children taking ARTs in sub Saharan Africa varies greatly 29-98%(9), with some countries reported higher adherence over the other (10–13). This can be attributed to the diversity of methods being used to assess adherence where self-reported measures are the commonest employed amongst low resource settings and thus have a tendency to overestimate adherence compared to the objective methods. It has been shown in these settings, that more accurate assessment of ART adherence can be obtained when two or more methods are employed to assess adherence(14).

Adherence has seen to be complex amongst children as compared to adults with different pressing issues affecting it, as they have to rely on the caregivers and their wellbeing to obtain drugs and face multiple challenges in social, health system and drug related aspects. Poor socio-economic circumstances including age of caregivers, poverty, single marital status and stigma were seen to be associated with poor adherence among countries like Cape Town, Uganda, Ethiopia, Nigeria and Northern Tanzania (10,12,15–17). Various studies have related poor adherence to caregiver factors, as seen with children who are under the care of non-parental guardians and single parents or busy care-givers(18–21).

In some countries, the regimen and health system related factors have seen to pose a barrier for good adherence, whereby complexity, tediousness of the pediatric regimen were noted in

some places like South India and Ethiopia(10,22) and drug side effects and nausea were seen to impede adherence in Northern Tanzania and Jamaica, (17,19).

Enhancing adherence and understanding its barriers in the local context is crucial and must be an ongoing process to ensure ART success. Data amongst children and early teenagers is lacking in Tanzania and specifically in Dar es Salaam. This study aims to cater for this gap, and assess the magnitude and attributes of ART adherence in this special group in order to design appropriate interventions to improve, or maintain optimal adherence levels.

MATERIALS AND METHODS

Study design and area

This was a hospital based descriptive cross-sectional study conducted in three sites within the city centre of Dar-es-Salaam; Muhimbili National Hospital (MNH), Temeke Regional Referral Centre (TRRH) and Infectious Disease Centre-DarDar Paediatric Clinic (IDC-DPP) HIV care and treatment clinics catering for children 0-14 years with their caregivers' and have regular follow-ups' in accordance with the national guidelines for management of HIV/AIDS.

Participants and eligibility criteria

The study participants were confirmed HIV positive children (1-14 years) already initiated on anti-retroviral therapy for atleast six months and attending regular follow up clinic with their caregivers at the three afore-mentioned CTC sites. Those on presumptive treatment were excluded.

We screened the child's clinical hospital records, CTC 1 and 2 cards for confirmation of the diagnosis of HIV and the duration of treatment and follow up at the CTC. Pharmacy records were checked for confirmation of regular attendance for drug pick up. Participants and their caregivers were consecutively enrolled until the sample size was achieved, if they fulfilled the inclusion criteria and consented.

Quota sampling calculation technique was used to obtain the number of participants from each respective CTC sites to ensure even distribution of the participants. This was based on the total number of patients registered at the CTC clinic.

Variables and data measurement

The variables measured in this study included: adherence level, factors associated with ART adherence after being on ART for at least six months. For uniformity, responses were obtained by the care-giver wherever possible.

Measures of adherence and outcomes

Three measurement tools were used to assess adherence for all participants: four-day self-recall report, one-month self-recall report (using the visual analogue scale) and number of scheduled clinic appointments missed. For each of these measures, greater than or equal to 95% responses were considered as good adherence, while less than that were taken as poor adherence(23).

4-day self-recall report: Care-givers were asked to recall if they had missed any dose over the last 4 days. If they reported missing more than one dose, it was considered as poor adherence <95%. If they reported one or no missed doses it was considered good adherence (≥95%).

1 month-self recall report (Visual Analogue Scale): Care-givers were asked to mark on a calibrated line scaled 1-10, as a reflection of the way they administered the medications to the child over the last one month. Thereafter, the mark was translated into percentage to estimate their drug administration estimate over the entire month. If the mark was below 9.5 (<95%) it was considered as poor adherence. If marked was at or above 9.5 (≥95%) it was taken as good adherence.

The number of missed clinic appointments: Records of the number of visits scheduled over the past 6 months were asked and confirmed from the CTC card and then the number of visits missed/ delayed according to the clinic's allowed time for delay, were checked from their CTC card and recorded accordingly. Each CTC had a maximum tolerance for delay where they were provided adequate drugs for that delay period. If there was no attendance beyond the allowed delay period, it was considered a missed visit. If the participant missed his/her scheduled appointment but attended it within the maximum allowed by the CTC, it was not considered a delay. This was further counter-checked with the pharmacies' refill records to ensure the drugs were picked up. If the participant missed more than one scheduled clinic visit it was considered as poor adherence (<95%).

Thus after documenting all the three adherence measures, Overall good adherence was qualified when the participant did well (≥95%) in all three adherence measures. If he/she

performed less than 95% in any one adherence measure, he/she was qualified as having poor overall adherence.

Factors affecting adherence

Factors affecting ART adherence taken in this study were: socio-demographic factors, participant related factors, drug regimen related factors and health system related factors, obtained from interviewing the caregiver.

Statistical analysis

Data was analyzed using SPSS software version 20. Quality control checks were assured during data entry process. Measures of central tendency were used to summarize discrete data. Charts, graphs and tables were used to display categorical data. Overall level of ART drug adherence was calculated and Contingency tables were constructed for Univariate analysis, to explore factors associated with levels of ART adherence. Chi-square test was used to determine associations between the dependent and independent variables. Level of significant association was set at $p<0.05$.

Univariate and multivariate logistic regression models were used to determine Odds ratios and p values for different factors associated with adherence. Only those factors whose Odds ratios had p values<0.2 on univariate analysis were further adjusted for in grouped manner on multivariate analysis. Adjusted Odds ratios with p values<0.05 on multivariate analysis were considered significant.

RESULTS

Recruitment and sampling of participants

There were a total of 830 attendants in the HIV care and treatment clinics among the three chosen centres during the study period. 476 of them failed the inclusion criteria and an additional 21 of those eligible, refused to consent as displayed on the flow diagram (Figure 1). A total of 333 participants met the inclusion criteria and were enrolled into the study and analysed. The non-response rate was found to be 6%.

ART Adherence among HIV infected children

Amongst a total of 333 participants that were recruited, approximately 60% were noted to have good adherence as shown in Figure 2.

Figure 3 displays the ART adherence level by each method of adherence assessment.

The four-day self-recall and missed clinic visits had an almost similar adherence rate of 93-94% as compared to the one-month self-recall report using VAS which showed a considerably lower level of adherence (61.6%). However, this was concordant with the overall adherence level.

Socio-demographic characteristics of the study participants

Among the 333 participants, the median age was 11 years with an interquartile range of 7-13 years. A slightly higher proportion of the participants were male (53.2%) and more of the participants were in the adolescent age group (10-14yrs) (61.3 %), and majority were attending school (90.7%).

Most of the participants (82.9%), had at least one parent and for most they were their biological parent (73.6%). About one third of caregivers (34.5%) were unemployed and the majority of caregivers (75%) had an average gross monthly income of less than 100,000 Tzs. Caregivers were aged between 17 and 75 with a median age of 40 years (IQR 34-46yrs), of which 70% were above 35 years of age.

Table 1: Participant and caregiver socio-demographic characteristics

Variable	Frequency (%) N=333
**Sex of Child**	
Male	177 (53.2)
Female	156 (46.8)
**Child Age Group (years)**	
Pre-Adolescent (1-9)	129 (38.7)
Adolescent(10-14)	204 (61.3)
**Child Schooling Status**	
Attending school	302 (90.7)
Not attending school	31 (9.3)
**Parental Status**	
Single/Both Parents	276 (82.9)
Non-parental caregiver	57 (17.1)
**Relation of Caregiver**	
Biological Parent	245 (73.6)
Non-Biological Parent	88 (26.4)
**Caregiver Age Group**	
< 25 years	26 (7.8)
25 – 35 years	74 (22.2)
> 35 years	233 (70.0)
**Caregiver Marital Status**	
Married/Cohabiting	177 (53.2)
Single/Divorced/Widowed	156 (46.8)
**Employment Status**	
Employed	218 (65.5)
Unemployed	115 (34.5)
**Monthly Income**	
Less than 100,000	250 (75.1)
100000 and above	83 (24.9)

Factors associated with ART adherence

Socio-demographics factors associated with ART adherence

Table 2 shows the univariate analysis of the selected socio-demographic characteristics in relation to ART adherence. The only factor noted to be associated with ART adherence was the caregivers' age group. Children whose caregivers were less than 25 years were two times more likely to have poor adherence [OR= 2.636, 95% CI 1.146-6.066 p= 0.023] when compared to children whose caregivers were over 25 years.

On further investigation about these younger aged caregivers, it was noted six amongst the twenty six were older siblings caring for their younger ones in the absence of their parents and the remaining were young mothers.

Table 2: Socio-demographic characteristics associated with ART adherence

Variable	Adherence		Total (N = 333)	OR (95% CI)	P-Value
	Poor (%) (N=134)	Good (%) (N=199)			
**Gender**					
Male	74(41.8)	103(58.2)	177	1.150 (0.741 – 1.784)	0.534
Female	60(38.5)	96(61.5)	156		
**Child Age Group**					
Pre-Adolescent (1-9yrs)	45(34.9)	84(65.1)	129	0.692(0.439 – 1.092)	0.113
Adolescent (10-14yrs)	89(43.6)	115(56.4)	204		
**Child Schooling Status**					
Attending school	121(40.1)	181(59.9)	302	0.926(0.437 – 1.959)	0.840
Not attending school	13(41.9)	18(58.1)	31		
**Parental Status**					
Single/Both Parents	110 (39.0)	166(60.1)	276	0.911 (0.511 – 1.625)	0.762
Non-parental caregiver	24(42.1)	33(57.9)	57		
**Relation of Caregiver**					
Biological Parent	95(38.8)	150(61.2)	245	0.796(0.486 – 1.303)	0.364
Non-Biological Parent	39(44.3)	49(55.7)	88		
**Caregiver Age Group**					
< 25 years	16(61.5)	10(38.5)	26	2.636 (1.146-6.066)	**0.023**
25 – 35 years	30(40.5)	44(59.5)	74	1.123 (0.658-1.917)	0.669
> 35 years	88(37.8)	145(62.2)	233	1 *Reference variable*	
**Caregiver Marital Status**					
Married/Cohabiting	71(40.1)	106(59.9)	177	0.989 (0.638 – 1.534)	0.960
Single/Divorced/Widowed	63(40.4)	93(59.6)	156		
**Employment Status**					
Employed	86(39.4)	132(60.6)	218	0.909 (0.574 – 1.440)	0.685
Unemployed	48(41.7)	67(58.3)	115		
**Monthly Income**					
Less than 100,000	101(40.4)	149(59.6)	250	1.027(0.619 – 1.705)	0.918
100000 and above	33(39.8)	50(60.2)	83		

Participant factors associated with ART adherence

Participant factors that were associated with poor ART adherence on univariate analysis, were found to be children having an inter-current illness which was eighteen times more likely to be associated with poor adherence (OR=18.3; 95% CI 4.214 – 79.513; p<0.001) and those who had adequate access to food, were two and a half times more likely to be associated with poor adhe[illegible]ce (OR=2.507; 95% CI 1.452 – 4.330; p=0.001) However, caregiver's belief in the effectiveness of antiretroviral therapy and their children having a

lower HIV clinical stage (1-2), were less likely to be associated with poor adherence (OR=0.118; 95% CI 0.044 – 0.318; p<0.001) (OR= 0.519; 95% CI 0.310 – 0.870; p=0.012) respectively and these were found to be statistically significant as seen in Table 3.

Table 3: Participant characteristics associated with ART Adherence

Variable	Adherence		Total (N = 333)	OR (95% CI)	P-Value
	Poor (%) (N=134)	Good (%) (N=199)			
**HIV Status disclosure**					
Yes	66(45.2)	80(54.6)	146	1.444(0.928 – 2.245)	0.103
No	68(36.4)	119(63.6)	187		
**Experiencing Stigma**					
Yes	121(40.5)	178(59.5)	299	1.098(0.530 – 2.277)	0.801
No	13(38.2)	21(61.8)	34		
**Child Inter-current illness**					
Yes	21(91.3)	2(8.7)	23	18.3(4.214 – 79.513)	**<0.001**
No	113(36.5)	197(63.5)	310		
**Availability of Adequate Food**					
Yes	39(58.2)	28(41.8)	67	2.507(1.452 – 4.330)	**0.001**
No	95(35.7)	171(64.3)	266		
**Believes on ART Effectiveness**					
Yes	110(36.2)	194(63.8)	304	0.118(0.044 – 0.318)	**<0.001**
No	24(82.8)	5(17.2)	29		
**Clinical Stage**					
Stage 1 – 2	94(36.6)	163(63.4)	257	0.519(0.310 – 0.870)	**0.012**
Stage 3 – 4	40(52.6)	36(47.4)	76		

Regimen related and Health system related factors associated with ART adherence

Tables 4 and 5 demonstrate the selected regimen related and health facility factors assessed in this study. None of these factors were significantly associated with poor adherence.

Table 4: Drug regimen related characteristics associated with ART Adherence

Variable	Adherence		Total (N = 333)	OR (95% CI)	P-Value
	**Poor (%) (N=134)**	**Good (%) (N=199)**			
**Type of ART Regimen**					
Lopinavir/ritonavir based	27(37.5)	45(62.5)	72	*1 Reference Variable*	
Efavirenz based	26(32.5)	54(67.5)	80	0.802(0.411 – 1.565)	0.519
Nevirapine based	81(44.2)	100(55.2)	181	1.350(0.771 – 2.363)	0.293
**ART Duration Use**					
Less than 2 years	12(36.4)	21(63.6)	33	0.834(0.396 – 1.757)	0.633
2 years and more	122(40.7)	178(59.3)	300		
**Number of pills per dose**					
1-pill	30(45.5)	36(54.5)	66	1.306 (0.759 – 2.249)	0.335
More than 1 pill	104(39.0)	163(61.0)	267		
**Person administering dose**					
Child him/herself only	10(55.6)	8(44.4)	18	1.846(0.692 – 4.927)	0.221
Caregiver only	59(38.3)	95(61.7)	154	0.917(0.583 – 1.442)	0.708
Child and caregiver	65(40.4)	96(59.6)	161	*1 Reference Variable*	
**Side effects Experienced**					
Yes	19(48.7)	20(51.3)	39	1.479(0.757 – 2.890)	0.253
No	115(39.1)	179(60.9)	294		

Table 5: Health system related characteristics associated with ART Adherence

Variable	Adherence		Total (N = 333)	OR (95% CI)	P-Value
	**Poor (%) (N=134)**	**Good (%) (N=199)**			
**Drug Change due to Unavailability**					
Yes	39(39.8)	59(60.2)	98	0.974(0.602 – 1.576)	0.915
No	95(40.4)	140(59.6)	235		
**Adequate Drug Refill**					
Yes	107(41.5)	151(58.5)	258	1.260(0.740 – 2.146)	0.395
No	27(36.0)	48(64.0)	75		
**Difficulty Accessing Health Facility**					
Yes	76(41.8)	106(58.2)	182	1.15(0.740 – 1.787)	0.535
No	58(38.4)	93(61.6)	151		
**Clinic Time Convenient**					
Yes	57(35.0)	106(65.0)	163	0.649(0.418 – 1.010)	0.055
No	77(45.3)	93(54.7)	170		

**Counseled on importance of Adherence**					
Yes	130(39.6)	198(60.4)	328	0.164(0.018 – 1.485)	0.108
No	4(80.0)	1(20.0)	5		

Independent factors associated with poor ART adherence

The possible factors that were seen to be associated to ART adherence on univariate analysis were further analysed for confounder effect. Multivariate analysis results for those factors and additionally others with p<0.2 are displayed in table 6. It was seen that the factors which remained to be significantly associated with poor adherence, were younger age group care-givers < 25 years and child having an inter-current illness (OR=3.520; 95% CI 1.471 – 8.422; p=0.005) and (OR=10.869; 95% CI 2.340 – 50.489; p=0.002) respectively.

On the other hand, a caregiver's belief in ART effectiveness and a child having a lower clinical stage were less likely to be associated with poor adherence (OR= 0.182; 95% CI 0.055 – 0.599; p=0.005) and (OR= 0.507; 95% CI 0.287 – 0.893; p=0.019) respectively.

Availability of adequate food lost statistical significance on multivariate analysis. Moreover, Child age group, HIV disclosure, clinic timings and counselling were not found to be associated with poor ART adherence.

Table 6: Independent factors associated with poor ART adherence

Variables	aOR (95% CI)	P-Value
**Child Age Group**		
Pre-Adolescent	0.848 (0.453 – 1.587)	0.607
Adolescent	1	
**Care Giver Age Group**		
< 25 years	3.520 (1.471 – 8.422)	**0.005**
25 – 35 years	1.431 (0.781 – 2.619)	0.246
> 35 years	1	
**HIV Status Disclosed to Child**		
Yes	1.303 (0.717 – 2.367)	0.385
No	1	
**Child inter-current Illness**		
Yes	10.869 (2.340 – 50.489)	**0.002**
No	1	

**Availability of Adequate Food**		
Yes	1.574 (0.830 – 2.984)	0.165
No	1	
**Believes on Effectiveness of ART**		
Yes	0.182 (0.055 – 0.599)	**0.005**
No	1	
**Clinical Stage**		
Stage 1 – 2	0.507 (0.287 – 0.893)	**0.019**
Stage 3 – 4	1	
**Clinic Timing Convenient**		
Yes	0.711 (0.430 – 1.177)	0.185
No	1	
**Counseled on Importance of Adherence**		
Yes	1.448 (0.106 – 19.706)	0.781
No		

Caregiver reasons for missing doses in HIV infected children

For those caregivers who reported missing even one pill, reasons were explored and some of the main challenges cited by them were; forgetting to take pills daily (41%) and high pill burden (21%) as the predominating factors followed by busy schedule- interrupting drug administration (11%) and long waiting hours at the clinic (9%). These, among the other cited reasons are shown in figure 4.

DISCUSSION

Adherence level among HIV infected children

Adherence to ARTs among children is crucial to ensure treatment success. Poor adherence results into sub-optimal drug levels making it less effective in suppressing viral replication resulting in treatment failure. As a result, viral load increases (virological failure), CD4 declines (immunological failure) and opportunistic infections subsequently occur (clinical failure) after a period of incomplete viral suppression(24).

The overall ART adherence of approximately 60%, is in keeping with a study done in Nigeria in 2015, and close to that reported in rural Tanzania, during the same year 65.6% and 70% respectively(16,20). A systematic review of ART adherence in low and middle income

countries in 2008 found a range in adherence estimates from 49% to 100% using different adherence measures(25).

In contrast to our findings, adherence was reported to be higher ranging from 72% to 94% among the last two decades in other sub-Saharan countries including; Ethiopia, Tanzania, Malawi and Uganda, with most studies reporting>90% ART adherence by self-recall means(10–13). Similarly, a single centred study done in Northern Tanzania, subjected to the three methods, reported a significantly lower adherence level of 24.6% among HIV infected children(17). Our comparatively higher adherence estimates despite multiple methods, could be explained by the considerable improvement in CTC services, free ART access and improved adherence counselling over the past half-decade.

The possible explanation of the exceptionally high contrasting results, obtained by the other sub-Saharan countries, could be due to their use of short term recall methods to assess adherence. In fact, in this study, using the four day recall method alone to measure adherence would have shown similar high adherence of 93.4% as these studies. This method, when used alone, is known to over-estimate ART adherence, especially if caregivers are the ones reporting, due to their vulnerability to social desirability and over-reporting. These biases could result in falsely inflated adherence estimates in the actual situation(25). While the VAS score is subjected to recall bias, it is the only measure which may capture the participant's own perception of his/her adherence, a measure of self-evaluation and may actually affect the outcome of future adherence and thus is an important measure that should not be omitted.

Factors affecting ART adherence in children

Socio-demographic characteristics affecting ART adherence

Multivariate logistic regression analysis indicated that the age of caregiver was an independent factor associated with ART adherence. Children, whose caregivers' were less than 25 years, were significantly found to be associated with poor adherence. These findings were similar to those reported from Kano (Nigeria) and Mekelle (Ethiopia)(10,16). However, caregivers' marital status and their economic condition did not seem to have an effect in this study like it did in the aforementioned surveys. Similarly, findings from KCMC in Northern Tanzania, suggested caregivers' low monthly income was associated with poorer adherence(17). The caregivers' relationship to the child had little effect on adherence status

of the child, unlike the findings reported from a region in rural part of Tanzania-Ifakara in 2015 by Nyogea *et al* from Tanzania where step mothers contributed to poor adherence(20).

A child's optimal adherence strongly relies on the caregiver's commitment; the younger age group are more likely to be less responsible towards this entrustment than their counterparts, who may have a better understanding of the dynamics of disease and health and thus, deserve more focused attention on adherence counselling and may entail health providers to identify older household members to be involved hand in hand in the care of the child.

Regimen related and health facility related factors affecting adherence

The study took place in centres with trained and motivated staff, where all the facilities provided care which was in line with the national ART scale up policy, by using simple once/twice daily regimens, fixed dose combinations when available and constant supply at all times. Due to the fully funded HIV CTC services, participants no longer faced the regimen related and healthcare related barriers to ART adherence including: cost of drugs, difficulty in accessing healthcare facilities, inadequate refills, unqualified regimen changes and pill burden. Therefore, these factors were not found to significantly influence ART adherence in this study; which was in line with other studies in Sub-Saharan African countries with similar setups in providing ART services(13,17,26–28).

Furthermore, counselling has been recognised as a key component to successful ART use and has been incooperated into the HIV care programme. In this study, lack of counselling was not associated with poor adherence unlike previous study done in Tanzania in 2004-2005, where continued counselling was not yet recognised, and was reported that only 21% of patients saw a counsellor on the day of their interview(27). With the improvement in CTC services and regular laboratory monitoring tests; HIV viral load and CD4 count motivate adherence, as patients are now aware that their laboratory results are improving.

In this study, less than one-fifth of the participants cited ART side effects as a constraint to ART use, and this was similar to a study done in Uganda and Botswana(12,29) and was contrary to the findings from northern Tanzania, Ethiopia and Jamaica(10,17,19) where, the drug side effects posed a barrier to optimal ART adherence and this could be explained by the fact that most of the participants in this study had been on ARTs for more than two years and these effects reduce with time and patients are counselled on and adapt strategies to overcome its adversities, another plausible explanation could be change in drug brands whereby the newer brands have less side-effects.

Participant factors affecting ART adherence

This study highlighted that, the most significant barriers to good ART adherence were those related to the participant themselves; Child's inter-current illness, availability of adequate food, belief in the effectiveness of ARTs and HIV clinical stage. In few studies, some but not all these factors were found to be obstacles for ART. A study done in Nigeria in 2010, reported child's inter-current illness and non-belief in ARTs; while a study in rural Tanzania (Ifakara) found preferring herbal medicine and experiencing stigma as barriers for ART adherence(16,20). On the contrary, a study in urban Malawi, Ethiopia and northern Tanzania found no significant relation of ART adherence with a child's WHO clinical stage or caregivers' belief in ART effectiveness(13,17,30). Non-disclosure and stigma were not found to affect adherence in this study as it did in earlier studies(12,29). The most plausible reason for this difference may be due to improved awareness, acceptance of HIV/AIDS and encouragement of timely child disclosure.

To explain our findings, we speculate; in Tanzania while the knowledge of HIV/AIDS and its treatment is overall good, beliefs of an existing super natural power that may have afflicted an HIV infected person still exists, leading to preference for alternative healing (herbs, spiritual medicine) which may hinder ART use. The findings point to a possible serious gap in caregivers awareness of health implications of non-adherence during illness and advanced stage, coupled with a false belief that taking ARVs with other drugs or during concomitant illness could result in serious adversities. Further studies on caregivers' belief aspects need to be carried out to confirm this finding.

Surprisingly in this study, availability of adequate food was found to be associated with poor adherence, similar to findings from Addis Ababa(30), however this lost significance on multivariate analysis. It is difficult to speculate about this association, as several studies have deemed nutrition availability and support as an integral part of successful HIV care and treatment(31–33) losing its significance on further analysis verified its confounder effect. Moreover, under-nutrition was noted among less than one-fifth of the participants (17%) signifying that it poses less of a problem in the recent times than it did around a decade earlier in Tanzania(27) and Ethiopia(34). The possible explanation for this could be that most of these patients were not ART naïve and had been attending the clinic and receiving counselling; which may have improved their nutritional status. Furthermore, nutrition is not entirely dependent on adherence to ARVs, rather is determined by multiple factors like

educational and socioeconomic status, family size, dietary diversity and other comorbid conditions(33,35,36).

Caregivers' reasons for missing medications

The main problems cited by the caregivers, who were responsible for a missed dose in this study were: forgetfulness, high pill burden, busy schedule and long waiting hours at the clinic. Some, but not all of these factors, were reported in other studies from similar resource limited countries like Ethiopia-Mekelle which found the pill burden and a survey in Addis Ababa found forgetfulness (23.5%)(34) as barriers to observing adherence. A study done in Nigeria also reported similar findings as ours; forgetfulness (59.5%), travel away from home/busy (21.4%) and child inter-current illness (14.3%)(16).

A multi-centre study in Tanzania reported long waiting hours at the clinic to be a hinderance to effective adherence(27) which was similar to findings in this study. This is possibly because some HIV clinics operated only once a week for children. In addition to that, with current provision of free drugs, the demand is higher and more prone to having longer waiting hours, unlike previous times where patients had to pay for their drugs. This can lead to patient dissatisfaction and default from treatment(37). The pill burden was observed to be high among most of the participants of this study and this could be due to the deficiency of paediatric fixed dose combinations available for every ART regime, as is available for adults- as a single pill taken once a day.

On the contrary, earlier studies reported explanations of suboptimal adherence being cost of transportation, difficulty accessing health facilities, cost of medication and lack of adequate counselling(27,38). During those times, HIV care was not entirely free and widely distributed and accessible. Patients had to bear some costs of their treatment, including buying their drugs and paying for lab tests. Currently with support funding from PEPFAR, HIV drugs and services is cost-free and clinics and transport services are easily accessed and thus not reported to be problematic.

The strengths of this study were that it was conducted in three centres in the region including primary and tertiary level health facilities, serving participants from most districts. Therefore the data obtained is reflective of the actual situation of adherence in our surroundings. Furthermore, the parameters used to assess adherence were practical and easily employed in any low resource clinical setting. Three separate measures were incooperated, to improve the reliability of the assessment, and thus remove the bias of one measure over the other.

This study should be interpreted with the following limitations in mind: Cross-sectional studies of this nature are unable to capture trends in adherence, which are known to change over time, thus are limited in drawing conclusions regarding causal effect relationship of potential factors, a longitudinal component may be the most appropriate for this regard. In addition, classification of different adherence cut off points, adherence measures and study designs used in different studies may not be perfect to compare and contrast findings meticulously. With regards to the parameters used to assess adherence, they are liable to be affected by recall bias and social desirability bias and these may have over-estimated good adherence. However, efforts were made to mitigate them by employing three different methods, whereby, the 4 day recall method has nearly no recall bias and the clinic attendance method is highly objective and concrete. The one month recall method is actually the most accurate depiction of the care givers' own perception of the child's adherence and has implications on his/her future adherence pattern and is thus an important parameter that should not be excluded.

In conclusion, good adherence was noted only among 60% of the children attending HIV CTC clinics in Dar es Salaam. The factors influencing poor adherence were; younger age of caregivers and a child having inter-current illness whilst, belief in effectiveness of ARTs and a child in the initial HIV clinical stages fostered good adherence. The most cited caregiver related reasons for missing drugs were; forgetfulness, pill burden, busy schedule and long waiting hours at clinic.

Thus we recommend the following; health workers should assess for ART adherence and routinely do adherence counseling at each encounter with the caregiver, whereby these simple adherence tools should be in-cooperated, in order to identify and detect poor adherence with more focus on child-centered adherence counseling strategies so as to further improve adherence in children in order to achieve 90-90-90 target.

Healthcare providers should also seek to enlist older caregivers above the age of 25 years in the house-hold as treatment supporters for each child. The may team up with the younger caregivers to enhance ART adherence among children. Caregivers' should be counseled on the need to ensure ART adherence, particularly in children with inter-current illness and advanced clinical stage because it is during these periods when the children mostly need their medication to overcome their illness. Furthermore, health education at point of service, like

during the daily mornings talks, about the effectiveness of ARVs' and alleviating the mythical beliefs' about the HIV/AIDS and its treatment in order to improve adherence.

Abbreviations

ART	-	Anti Retroviral Therapy
ARV	-	Anti Retroviral
AIDS	-	Acquired Immunodeficiency syndrome
CD4	-	Cluster of Differentiation 4
CTC	-	Care and Treatment Clinic
DPP	-	DARDAR Paediatric Program
HIV	-	Human Immunodeficiency Virus
IDC	-	Infectious Disease Centre
MNH	-	Muhimbili National Hospital
MUHAS	-	Muhimbili University of Health and Allied Sciences
PEPFAR	-	President's Emergency Plan for AIDS Relief
SPSS	-	Statistical Package of Social Science Software
SR-Recall	-	Self-Reported Recall
TRRH	-	Temeke Regional Referral Hospital
UNAIDS	-	Joint United Nations Programme on HIV and AIDS
VAS	-	Visual Analogue Scale

Acknowledgements

We would like to give special thanks to the caregivers' and their children with HIV/AIDS who participated in this research, the administrative, medical and nursing staff of the Muhimbili National hospital, IDC- DarDar Paediatric Program HIV Clinic and Temeke Regional Referral Hospital. This work would have never been possible without them. We would also like to thank Dr Sibtain Moledina and Candida Moshiro for their advice in the statistical aspects of this study.

Authors' contribution

FMM conceived and designed the study, collected the data and constructed the manuscript. EAN and HPM supervised and gave advice in the development of the piece. FMM and SMM jointly analysed the data.

Ethics approval and consent to participate

Ethical approval to conduct the study was obtained from Ethics Review Committee of Muhimbili University of Health and Allied Sciences and permission sought from the respective health facilities. A written informed consent was sought from the caregiver and a formal written assent was obtained from all children seven years and above who had the capacity to understand prior to recruitment.

Consent for publication

Not applicable

Competing interests

The authors declare they have no competing interests

Data availability

Yes

REFERENCES

1. UNAIDS. Global HIV & AIDS statistics. 2019. p. 1–2.
2. Delia M Rivera RWS. What are the statistics on pediatric HIV infection in sub-Saharan Africa? Medscape Pediatrics. 2018.
3. Carpenter CC, Cooper DA, Fischl MA, Gatell JM, Gazzard BG, Hammer SM, Hirsch MS, Jacobsen DM, Katzenstein DA, Montaner JS RD. Antiretroviral Therapy in Adults. JAMA. 2000 Jan 19;283(3):381–90.
4. Simoni JM, Montgomery A, Martin E, New M, Demas PA, Rana S. Adherence to Antiretroviral Therapy for Pediatric HIV Infection: A Qualitative Systematic Review With Recommendations for Research and Clinical Management. Pediatrics. 2007;119(6):e1371–83.
5. Gibb DM, Goodall RL, Giacomet V, Mcgee L, Compagnucci A, Lyall H PEN for T of ASC, Gibb DM, Goodall RL, Giacomet V, McGee L, Compagnucci A, et al. Adherence to prescribed antiretroviral therapy in human immunodeficiency virus-infected children in the PENTA 5 trial. Pediatr Infect Dis J. 2003 Jan;22(1):p 56-62.
6. Elise A, France AM, Louise WM, Bata D, François R, Roger S PM. Assessment of Adherence to Highly Active Antiretroviral Therapy in a Cohort of African HIV-Infected Children in Abidjan, Cote D'Ivoire. JAIDS J Acquir Immune Defic Syndr. 2005 Dec;40(4):498–500.
7. Ivers LC, Cullen KA, Freedberg KA, Block S, Coates J, Webb P. HIV/AIDS, Undernutrition, and Food Insecurity. Clin Infect Dis. 2009 Oct 1;49(7):1096–102.
8. Wakeham K, Harding R, Bamukama-Namakoola D, Levin J, Kissa J, Parkes-Ratanshi R, et al. Symptom Burden in HIV-Infected Adults at Time of HIV Diagnosis in Rural Uganda. J Palliat Med. 2010 Apr;13(4):375–80.
9. Sutcliff CG, Dijk JH Van, Bolton C, Persaud D, Moss WJ. Eff ectiveness of antiretroviral therapy among HIV-infected children in sub-Saharan Africa. Lancet Infect Dis. 2008;8(34):477–89.
10. Eticha T BL. Caregiver-reported adherence to antiretroviral therapy among HIV infected children in Mekelle, Ethiopia. BMC Pediatr. 2014;14(1):114.
11. Mghamba FW, Minzi OMS, Massawe A, Sasi P. BMC Pediatrics Full text Adherence to antiretroviral therapy among HIV infected children measured by caretaker report, medication return, and drug level in Dar Es Salaam, Tanzania. BMC Pediatr. 2013;13(1):95.

12. Nabukeera-Barungi N, Kalyesubula I, Kekitiinwa A, Byakika-Tusiime J, Musoke P. Adherence to antiretroviral therapy in children attending Mulago Hospital, Kampala. Ann Trop Paediatr. 2007 Jun 18;27(2):123–31.
13. Weigel R, Makwiza I, Nyirenda J, Chiunguzeni D, Phiri S, Theobald S. Supporting children to adhere to anti-retroviral therapy in urban Malawi: Multi method insights. BMC Pediatr. 2009;9:1–9.
14. Berg KM, Arnsten JH. Practical and Conceptual Challenges in Measuring Antiretroviral Adherence. JAIDS J Acquir Immune Defic Syndr. 2006 Dec 1;43(Supplement 1):S79–87.
15. Davies MA, Boulle A, Fakir T, Nuttall J EB. Adherence to antiretroviral therapy in young children in Cape Town, South Africa, measured by medication return and caregiver self-report: A prospective cohort study. BMC Pediatr. 2008;8(34):1–12.
16. Zubayr BM, Airede K, Ibrahim M, Hassan-Hanga F, Jumare J, Gambo MJ, Gebi U, Etiebett M, Bowman D, Dakum P BW. Adherence to Highly Active Antiretroviral Therapy among HIV-Infected Children in Kano, Nigeria. J Hum Virol Retrovirology. 2015;56:85.
17. Nsheha AH ak., Dow DE lizabet., Kapanda GE ric., Hamel BC arolu., Msuya LJ anuar. Adherence to antiretroviral therapy among HIV-infected children receiving care at Kilimanjaro Christian Medical Centre (KCMC), Northern Tanzania: A cross-sectional analytical study. Pan Afr Med J. 2014;17(1):238.
18. Pontali E. Facilitating Adherence to Highly Active Antiretroviral Therapy in Children with HIV Infection. Pediatr Drugs. 2005;7(3):137–49.
19. White YRG, Pierre RB, Steel-Duncan J, Palmer P, Evans-Gilbert T, Moore J, et al. Adherence to antiretroviral drug therapy in children with HIV/AIDS in Jamaica. West Indian Med J. 2008;57(3):231–7.
20. Nyogea D, Mtenga S, Henning L, Franzeck FC, Glass TR, Letang E, et al. Determinants of antiretroviral adherence among HIV positive children and teenagers in rural Tanzania: A mixed methods study. BMC Infect Dis. 2015;15(1):1–13.
21. Fokam J, Billong SC, Jogue F, Ndiang SMT, Motaze ACN, Paul KN, et al. Immuno-virological response and associated factors amongst HIV-1 vertically infected adolescents in Yaoundé-Cameroon. PLoS One. 2017;12(11):e0187566.
22. Paranthaman K, Kumarasamy N, Bella D, Webster P. Factors influencing adherence to anti-retroviral treatment in children with human immunodeficiency virus in South India – a qualitative study. AIDS Care. 2009 Aug 17;21(8):1025–31.

23. Eley B, Nuttall J, Davies MA, Smith L, Cowburn C, Buys H, et al. Initial experience of a public sector antiretroviral treatment programme for HIV-infected children and their infected parents. S Afr Med J. 2004;94(8):643–6.
24. Reddington C, Cohen J, Baldillo A, Toye M, Smith D, Kneut C, Demaria A, Bertolli J HH. Adherence to medication regimens among children with human immunodeficiency virus infection. Pediatr Infect Dis J. 2000;19(12):1148–53.
25. Vreeman RC, Wiehe SE, Pearce EC, Nyandiko WM. A Systematic Review of Pediatric Adherence to Antiretroviral Therapy in Low- and Middle-Income Countries. Vol. 27, The Pediatric Infectious Disease Journal. 2008. p. 686–91.
26. Biadgilign S, Deribew A, Amberbir A DK. Barriers and facilitators to antiretroviral medication adherence among HIV-infected paediatric patients in Ethiopia: A qualitative study. J Soc Asp HIV/AIDS. 2009;6(4).
27. Irunde H, Temu F, Maridadi J. A study on antiretroviral adherence in Tanzania: a pre-intervention perspective, 2005. From access to adherence: the challenges of antiretroviral treatment. 2006. p. 164.
28. Mukhtar-Yola M, Adeleke S, Gwarzo D, Ladan ZF. Preliminary investigation of adherence to antiretroviral therapy among children in Aminu Kano Teaching Hospital, Nigeria. African J AIDS Res. 2009 Sep;5(2):141–4.
29. Weiser S, Wolfe W, Bangsberg D, Thior I, Gilbert P. Barriers to Antiretroviral Adherence for Patients Living with HIV. J Acquir Immune Defic Syndr. 2003;34(3):281–8.
30. Biadgilign S, Deribew A, Amberbir A, Deribe K. Adherence to highly active antiretroviral therapy and its correlates among HIV infected pediatric patients in Ethiopia. BMC Pediatr. 2008;8(1):1–9.
31. Mangili A, Murman DH, Zampini AM, Wanke CA, Mayer KH. Nutrition and HIV Infection: Review of Weight Loss and Wasting in the Era of Highly Active Antiretroviral Therapy from the Nutrition for Healthy Living Cohort. Clin Infect Dis. 2006 Mar 15;42(6):836–42.
32. Villamor E, Misegades L, Fataki MR, Mbise RL, Fawzi WW. Child mortality in relation to HIV infection , nutritional status , and socio-economic background. Int J Epidemiol. 2005;34(1):61–8.
33. Sunguya BF, Poudel KC, Mlunde LB, Urassa DP, Yasuoka J, Jimba M. Poor nutrition status and associated feeding practices among HIV-positive children in a food secure region in tanzania: A call for tailored nutrition training. PLoS One. 2014;9(5):e98308.

34. Biadgilign S, Deribew A, Amberbir A, Deribe K. Barriers and facilitators to antiretroviral medication adherence among HIV-infected paediatric patients in Ethiopia: A qualitative study. Sahara J. 2009;6(4):148–54.
35. Pongou R, Ezzati M, Salomon JA. Household and community socioeconomic and environmental determinants of child nutritional status in Cameroon. BMC Public Health. 2006 Dec 17;6(1):98.
36. Barros FC, Victora CG, Scherpbier R, Gwatkin D. Socioeconomic inequities in the health and nutrition of children in low/middle income countries. Rev Saude Publica. 2010 Feb;44(1):1–16.
37. Phiri S, Weigel R, Hosseinipour M, Boxshall M NF. The Lighthouse- a centre for comprehensive HIV/AIDS treatment and care in Malawi. p. A Case study.
38. Byakika-Tusiime J, Oyugi JH, Tumwikirize WA, Katabira ET, Mugyenyi PN, Bangsberg DR. Adherence to HIV antiretroviral therapy in HIV+ Ugandan patients purchasing therapy. Int J STD AIDS. 2005 Jan 25;16(1):38–41.

Figure

Click here to access/download;Figure;List of figures for manuscript.docx

MAGNITUDE AND ATTRIBUTES OF ANTI-RETROVIRAL THRAPY ADHERENCE AMONG CHILDREN ATTENDING HIV CARE AND TREATMENT CLINICS IN DAR ES SALAAM, TANZANIA

List of figures attached

1. Figure 1: Flow diagram showing recruitment of participants
2. Figure 2: Overall Level of ART adherence among children 1-14 years
3. Figure 3: Adherence Level By each method of adherence assessment
4. Figure 4: Reasons for missing doses in HIV infected children

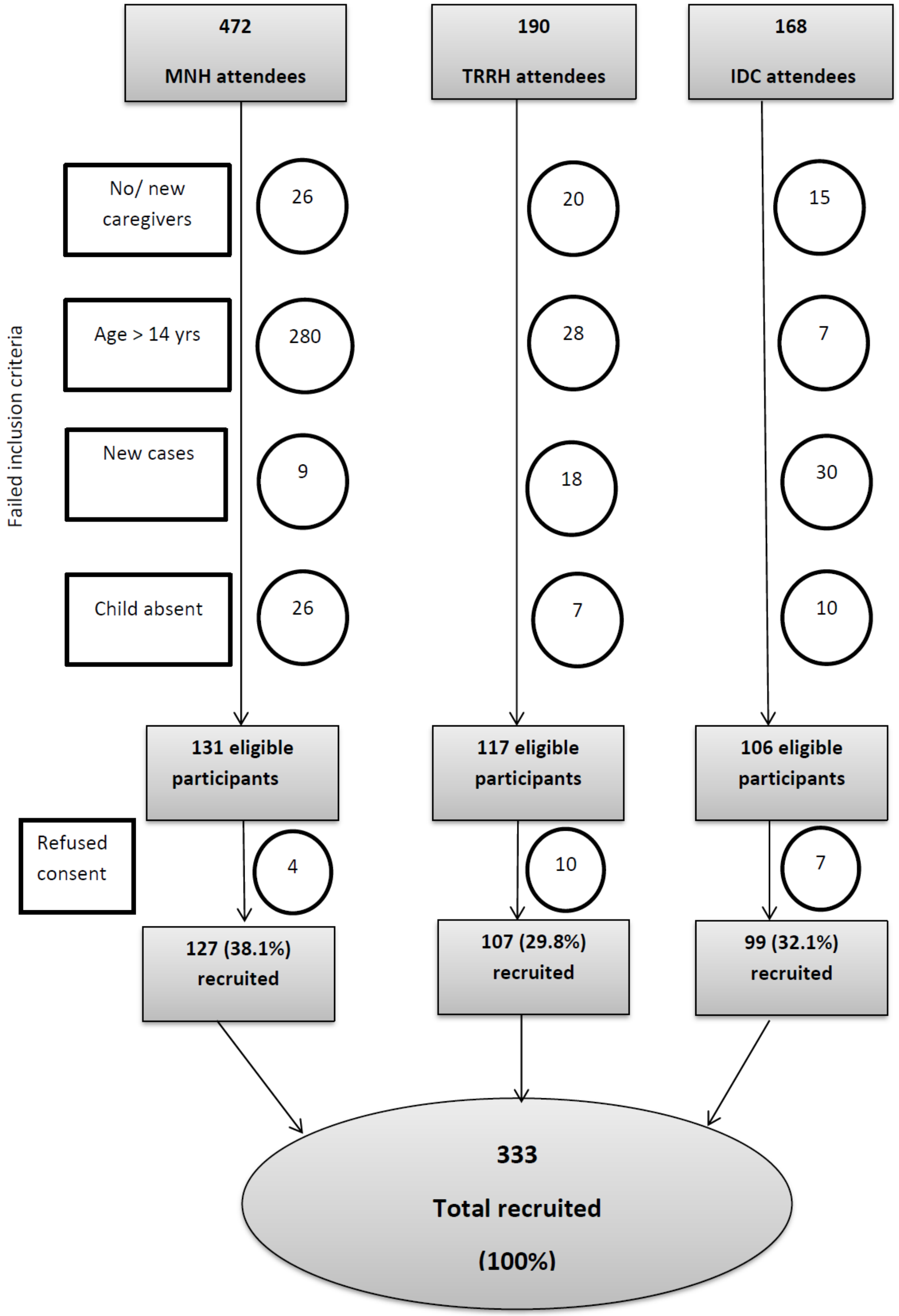

Figure 1: Flow diagram showing recruitment of participants

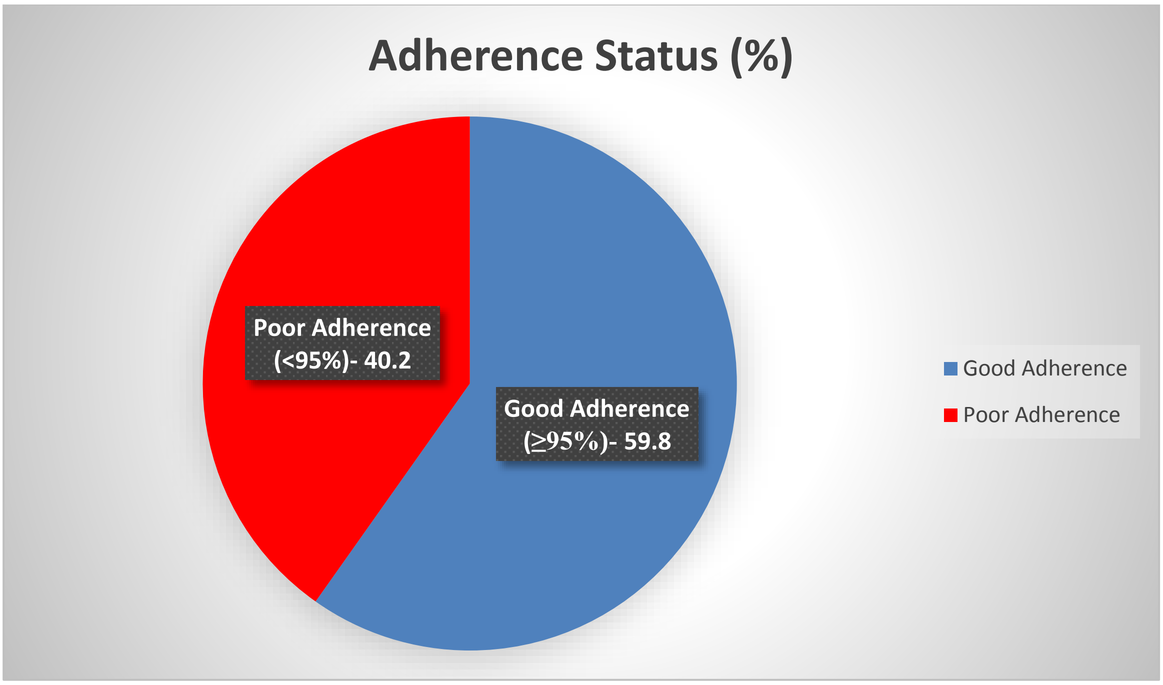

Figure 2: Overall Level of ART adherence among children 1-14 years

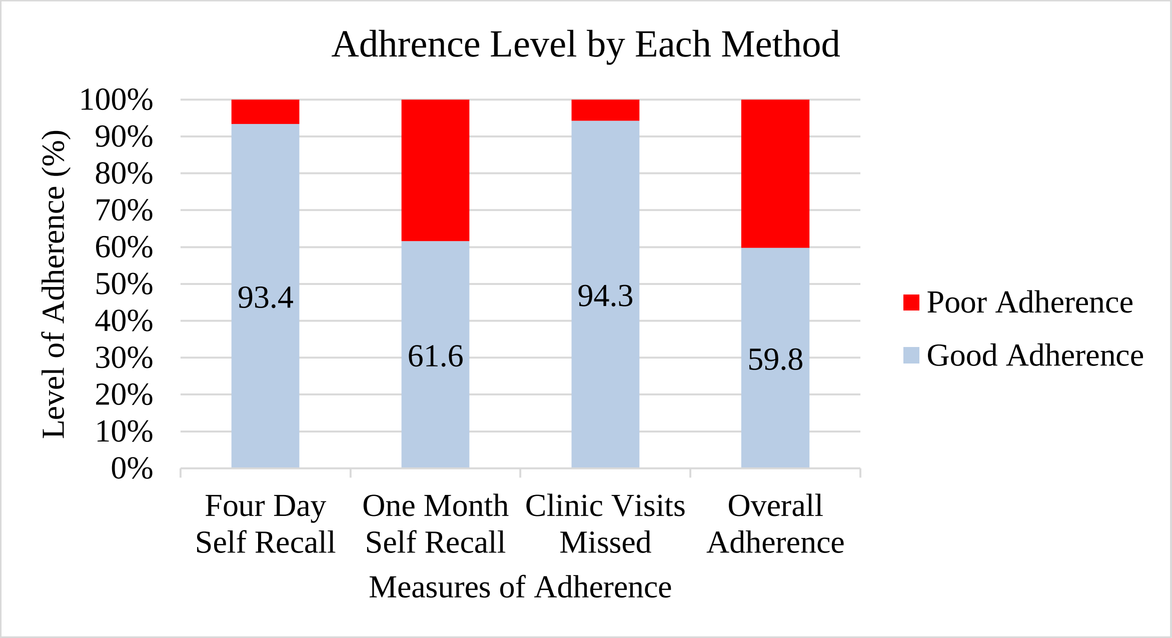

Figure 3: - Adherence Level By each method of adherence assessment

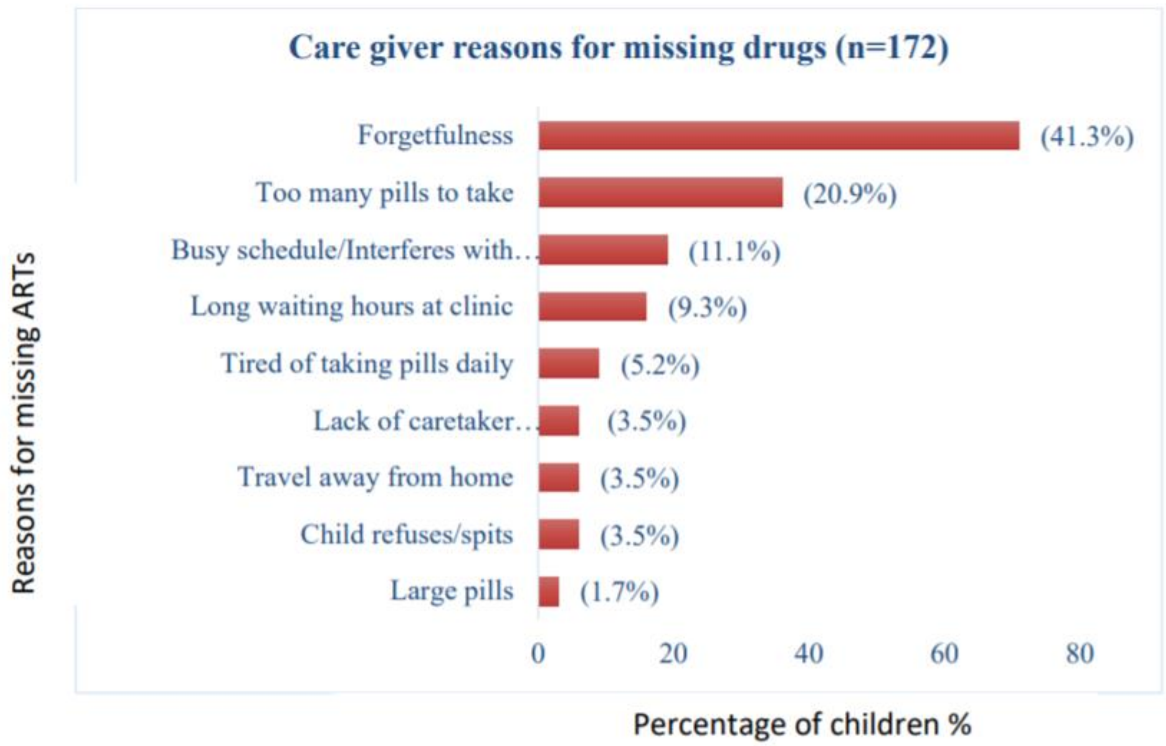

Figure 4- Reasons for missing doses in HIV infected children
