# Supplementary material for: Magnitude and associated factors of anti-retroviral therapy adherence among children attending HIV care and treatment clinics in Dar es Salaam, Tanzania
Source: PLoS One. 2022 Sep 30;17(9):e0275420. doi: 10.1371/journal.pone.0275420 (PMC9524636; doi:10.1371/journal.pone.0275420)
Supplement: Attachment — Submitted filename: comments for Plos One.docx [file pone.0275420.s002.docx]

**Title:**

1. Better to say “associated factors” rather than “attributes” in the title.
2. Originality: what makes your study unique from a similar study conducted in your country which is available with the following link?

https://bmcinfectdis.biomedcentral.com/articles/10.1186/s12879-015-0753-y?

**Key words:**

1. Please use key words rather than phrases like ART adherence, associated factors, children, Magnitude…..

**Abstract**

1. **Objectives:** the objective should be inline with your title i.e. “to assess magnitude and associated factors of ART adherence among children (1-14 years) attending HIV care …
2. **Result:** what are all the three measures**?** Please also approximate the odds ratios and confidence intervals to the nearest number and use only two-digit numbers like 3.520 → 3.5

**Introduction**

1. First begin by defining HIV/AIDS and adherence
2. Please also state the severity and magnitude of the problem (Poor ART adherence) in Tanzanian context as well.

**Methods and materials**

**Study design and area**

1. Your title includes 1-14 years but in study design you included 0-14 years. why?
2. In the paragraph you included children who had regular follow-up. Since you study adherence you have to include children who didn’t have also regular follow up. If you include clients who had regular follow up alone, it over estimates the magnitude of good adherence.

Participants and eligibility criteria

1. What is presumptive treatment? Is this recommended by WHO?
2. Why you used quota sampling? It is better to use probability sampling techniques in order to generalize your findings.
3. You used clinical hospital records. You have to set some exclusion criteria, if major variables are missed in the chart.

**Variables and data measurement**

1. Better to state your variables in categories of dependent (outcome variable) and independent (predictor) variables in paragraph form
2. Adherence measurement: adherence can be adherence to care (**clinical adherence**) meaning regular attendance of the patient according to given appointment, and **drug adherence** which means taking the drug according to instructions given by the providers. Your focus is adherence to drug (ART). Therefore, why you used missed clinic appointments as measure of adherence?
3. It is better to use WHO adherence scale by calculating percent (%) adherence and classify level of adherence as good, fair or poor. You can merge fair and poor based on your operational definition you stated.
4. In recall report of measuring adherence, how did you handle recall bias?

**Factors affecting adherence**

1. Since it is part of variables, it is better to write in variable section before measures of adherence and outcome.

**Statistical analysis:**

1. What is the importance of chi square test if you use odds ratio? Odds ratio shows measure as well as strength of association. Therefore, it is better to use odds ratio and logistic regression model.
2. In the logistic regression model, it is better to use bivariable and multivariable logistic regression rather than univariate regression

**Result:**

1. What is the importance of mentioning 830, 476 … if this population doesn’t meet inclusion criteria and not part of your study? Focus only on sample size.
2. Write sociodemographic characteristics before ART adherence among HIV infected children
3. What is the importance of writing table 4 and 5, if all of the variables are insignificant?
4. It is better to write the factors affecting adherence in 1 table

**Discussion**

1. Paragraph 1: please discuss only your pertinent findings. Paragraph 1 is not related to your study. Avoid it.
2. Please write similar concepts in 1 paragraph in brief and succinct way.
3. No need to write sub themes like “Factors affecting ART adherence in children…” in discussion. Please discuss the factors without subheadings.

**Conclusion:**

1. Conclude based on your objective

**Recommendation:**

2. Recommend based on your result

**Summary status:** Major revision
