# Supplementary material for: Magnitude and associated factors of anti-retroviral therapy adherence among children attending HIV care and treatment clinics in Dar es Salaam, Tanzania
Source: PLoS One. 2022 Sep 30;17(9):e0275420. doi: 10.1371/journal.pone.0275420 (PMC9524636; doi:10.1371/journal.pone.0275420)
Supplement: Attachment — Submitted filename: RESPONSE TO REVEIWERS.docx [file pone.0275420.s005.docx]

**RESPONSES TO REVIEWERS**

**REVIEWER 1**

**Title:**

1. Better to say “associated factors” rather than “attributes” in the title.

RESPONSE: CHANGED TO ASSOCIATED FACTORS

2. Originality: what makes your study unique from a similar study conducted in your country which is available with the following link?

https://bmcinfectdis.biomedcentral.com/articles/10.1186/s12879-015-0753-y?

RESPONSE: THIS STUDY WAS CONDUCTED IN A RURAL AREA IN IFAKARA IN 2015, IT WAS A MULTICENTRE STUDY WITH A SMALLER SAMPLE SIZE AND THAT MAY LIMIT GENERALIZABILITY EVEN IN OUR SETTING. A SINGLE MEASURE WAS USED TO ASSESS ADHERENCE. THEIR COHORT WAS A VERY WIDE AGE RANGE FROM 2-19 YEARS AND IT IS KNOWN THAT FACTORS AFFECTING ADHERENCE VARIES GREATLY AMONGST CHILDREN AND ADOLESCENTS.

ADHERENCE AND FACTORS AFFECTING IT, IS A DYNAMIC PROCESS THAT CHANGES OVER TIME AND NEEDS REGULAR ASSESMENT TO IMPROVE AND OVERCOME ITS BARRIERS, THAT WARRANTS US TO REVIEW THE OUTCOMES AS WE CHANGE AND IMPROVE OUR PRACTICES IN THE CARE OF THESE CHILDREN. FURTHERMORE, WE DID A MULTICENTRE STUDY, CHOSING DIFFERENT CADRES OF THE HEALTH SYSTEM FROM THE COUNTRYS NATIONAL REFERAL HOSPITAL TO A REGIONAL HOSPITAL CENTRE, TO A PRIMARY LEVEL OUT PATIENT CLINIC, THUS TRYING TO CAPTURE ADHERENCE AND ITS BARRIERS ACROSS ALL LEVELS IN THE MAIN CITY CENTRE OF THE COUNTRY. WE CHOSE TO TARGET THE YOUNGER AGED CHILDREN UNDER THE CARE OF CAREGIVERS RATHER THAN COMBINING THEM WITH ADOLESCENTS AS THEIR BARRIERS DIFFER GREATLY AND THUS OUR RESULTS ARE QUITE INDICATIVE OF THE SITUATION AFFECTING THIS PARTICULAR AGE GROUP.

**Key words:**

1. Please use key words rather than phrases like ART adherence, associated factors, children, Magnitude…..

RESPONSE: CHANGED TO KEYWORDS RATHER THAN PHRASES

**Abstract**

1. **Objectives:** the objective should be inline with your title i.e. “to assess magnitude and associated factors of ART adherence among children (1-14 years) attending HIV care …

RESPONSE: CHANGED TO THE ABOVE

2. **Result:** what are all the three measures**?** Please also approximate the odds ratios and confidence intervals to the nearest number and use only two-digit numbers like 3.520 → 3.5

RESPONSE: THE 3 MEASURES WERE DESCRIBED IN THE METHODS SECTION OF THE ABSTRACT AS FOUR DAY SELF-REPORT, ONE MONTH SELF-RECALL REPORT AND MISSED CLINIC APOINTEMENTS

HAVE APPROXIMATED THEM TO THE NEAREST DECIMAL PLACE

**Introduction**

1. First begin by defining HIV/AIDS and adherence

RESPONSE: HAVE INCLUDED THAT

2. Please also state the severity and magnitude of the problem (Poor ART adherence) in Tanzanian context as well.

RESPONSE: LIMITED STUDIES AVAILABLE FOR THAT ESPECIALLY AMONGST CHILDREN, HAVE INCLUDED WHAT WAS FOUND.

**Methods and materials**

**Study design and area**

1. Your title includes 1-14 years but in study design you included 0-14 years. why?

RESPONSE: MY APOLOGIES, THIS WAS A TYPO, HAVE CORRECTED IT.

2. In the paragraph you included children who had regular follow-up. Since you study adherence you have to include children who didn’t have also regular follow up. If you include clients who had regular follow up alone, it over estimates the magnitude of good adherence.

REPSONSE: THIS WAS A MISINTERPRETATION, PROBABLY A WRONG CHOICE OF WORDS ON MY SIDE; I MEANT CHILDREN WHO ARE ENROLLED IN THAT CLINIC AND SCHEDULED TO ATTEND THERE FOR REGULAR REFILLS AND FOLLOW UP VISITS. NOT THAT I CHOSE PATIENTS WHO REGULARLY ATTENDED. AMONGST THESE PATIENTS WERE THOSE WHO HAD POOR FOLLOW UPS AND THUS DETECTED ON MISSED CLINIC VISIT ADHERENCE MEASURE. HAVE CHANGED IT TO BE MORE CLEAR

Participants and eligibility criteria

1. What is presumptive treatment? Is this recommended by WHO?

PRESUMPTIVE TREATMENT IS PART OF OUR COUNTRIES NATIONAL HIV/AIDS TREATMENT GUIDELINES AND PART OF OUR PRACTICE WHERE INFANTS AND CHILDREN<18MONTHS BORN TO HIV POSITIVE MOTHERS ARE GIVEN A PRESUMPTIVE DIAGNOSIS OF SEVERE HIV INFECTION BASED ON CERTAIN CRITERIA IN ORDER NOT TO DELAY TREATMENT WHILE AWAITING CONFIRMATORY DNA PCR RESULTS. FURTHER DETAILS CAN BE FOUND FROM THE NATIONAL GUIDELINES FOR THE MANAGEMENT OF HIV/AIDS, 2017, CHAPTER 7, PAGE 111 ( Available in the references).

2. Why you used quota sampling? It is better to use probability sampling techniques in order to generalize your findings.

RESPONSE: QUOTA SAMPLING WAS USED AS WE WERE TIME BOUND AND HAD A TIGHT BUDGET.

3. You used clinical hospital records. You have to set some exclusion criteria, if major variables are missed in the chart.

RESPONSE: HOSPITAL RECORDS WERE NOT USED TO COLLECT DATA, AS DATA WAS COLLECTED FROM A STRUCTURED QUESTIONNAIRE DURING AN INTERVIEW PROCESS WITH THE CAREGIVER. THE RECORDS WERE ONLY USED TO CONFIRM THE DIAGNOSIS OF HIV, DRUG REGIMEN THEY ARE ON AND OTHER SUPPORTIVE INFORMATION. THIS ENSURED NO MISSING VARIABLES FOR OUR STUDY.

**Variables and data measurement**

1. Better to state your variables in categories of dependent (outcome variable) and independent (predictor) variables in paragraph form

RESPONSE: ADDED THAT AS A PARAGRAPH

2. Adherence measurement: adherence can be adherence to care (**clinical adherence**) meaning regular attendance of the patient according to given appointment, and **drug adherence** which means taking the drug according to instructions given by the providers. Your focus is adherence to drug (ART). Therefore, why you used missed clinic appointments as measure of adherence?

RESPONSE: AGREED, HOWEVER IN OUR SETUP DRUG REFILLS ARE PROVIDED DURING THESE SCHEDULED MONTHLY VISITS, THUS IF A PARTICIPANT DOESNOT ATTEND A CLINIC VISIT THEY WILL MISS A DRUG REFILL AND BE UNABLE TO TAKE THE DRUGS AS SCHEDULED AND INSTRUCTED BY THE PROVIDER AND THUS CONSIDERED AN OBJECTIVE WAY TO ASSESS DRUG ADHERENCE.

3. It is better to use WHO adherence scale by calculating percent (%) adherence and classify level of adherence as good, fair or poor. You can merge fair and poor based on your operational definition you stated.

RESPONSE: IN THIS STUDY, THE VISUAL ANALOGUE SCALE (VAS) WAS USED TO OBTAIN THE PERCENTAGE ADHERENCE AND CLASSIFIED AS POOR OR GOOD, THIS WAS CHOSEN AS THE CAREGIVER HAD TO OBSERVE THE SCALE AND MARK ON THEIR OWN NOT BEING INFLUENCED OR JUDGED FOR THE MARK THEY PUT THUS REDUCING THE EFFECT OF SOCIAL DESIRABILITY BIAS. FURTHER MORE IT GAVE A DEPICTION OF WHAT THEY PERCEIVED THEIR ADHERENCE WAS, WHICH IS IMPORTANT IN THEIR FUTURE ADHERENCE CHOICES. THIS SCALE HAS BEEN VALIDATED AMONGST STUDIES AND FOUND USEFUL IN RESEARCH AND CLINICAL SETTINGS.

4. In recall report of measuring adherence, how did you handle recall bias?

RESPONSE: IN ORDER TO MINIMISE THE RECALL BIAS WE CAREFULLY SELECTED THE QUESTIONS/ MEANS OF ASKING. USING THE VAS AND RECALLING OVER THE LAST MONTH WHICH WAS NOT TOO LONG AGO, AS IT HAS BEEN SHOWN RECALL BIAS IS PROBLEMATIC WHEN THE EVENT OCCURS LONG TIME AGO. FURTHER MORE WE COMBINED IT WITH SHORTER RECALL METHODS TO REDUCE THE RECALL ELEMENT AND MORE OBJECTIVE MISSED CLINIC VISIT.

**Factors affecting adherence**

1. Since it is part of variables, it is better to write in variable section before measures of adherence and outcome.

RESPONSE: I HAVE MOVED IT IN THE VARIABLES SECTION, BUT I FEEL ITS BETTER PUT AFTER MEASURES OF ADHERENCE AS ADHERENCE MEASURING IS THE FIRST OBJECTIVE AND FACTORS AFFECTING ADHERENCE IS THE SECOND OBJECTIVE.

**Statistical analysis:**

1. What is the importance of chi square test if you use odds ratio? Odds ratio shows measure as well as strength of association. Therefore, it is better to use odds ratio and logistic regression model.

RESPONSE: APOLOGIES, THAT WAS A TYPO ON MY SIDE, WE USED ODDS RATIO AND LOGISTIC REGRESSION MODELS. THANKS FOR NOTING THAT. I HAVE RECTIFIED THE TYPO.

2. In the logistic regression model, it is better to use bivariable and multivariable logistic regression rather than univariate regression

RESPONSE: AGREED, BIVARIATE AND MULTIVARIABLE LOGISTIC REGRESSION WAS DONE.

**Result:**

1. What is the importance of mentioning 830, 476 … if this population doesn’t meet inclusion criteria and not part of your study? Focus only on sample size.

RESPONSE: REMOVED THE FLOW DIAGRAM AND EXPLANATION ON RECRUITMENT.

2. Write sociodemographic characteristics before ART adherence among HIV infected children

RESPONSE: DONE

3. What is the importance of writing table 4 and 5, if all of the variables are insignificant?

RESPONSE: MY TAKE ON THIS WOULD DIFFER, THE STUDY OBJECTIVE WAS TO FIND OUT THE FACTORS AFFECTING ART ADHERENCE AMONG CHILDREN, IN TERMS OF THEIR SOCIO-DEMOGRAPHIC CHARACTERISTICS, HEALTH FACILITY/ DRUG RELATED FACTORS. IT WAS ONLY AFTER MULTIVARIABLE LOGISTIC REGRESSION IT LOST SIGNIFICANCE. I THINK ITS IMPORTANT TO SHOW THEY WERE SOUGHT, HOWEVER LOST SIGNIFICANCE AFTER REGRESSION ANALYSIS.

4. It is better to write the factors affecting adherence in 1 table

RESPONSE: TABLE 2,3,4,5 IS SHOWING BIVARIATE REGRESSION ANALYSIS OF THESE FACTORS. HOWEVER TABLE 6 IS ONLY DEPICTING THOSE FOUND SIGNIFICANT ON BIVARIATE ANALYSIS, BEING FURTHER ANALYSED IN MULTIVARIABLE LOGISTIC REGRESSION MODEL AND THUS COMING UP WITH THE MOST SIGNIFICANT FACTORS IN ONE TABLE.

**Discussion**

1. Paragraph 1: please discuss only your pertinent findings. Paragraph 1 is not related to your study. Avoid it.

RESPONSE: THANKS, REMOVED IT.

2. Please write similar concepts in 1 paragraph in brief and succinct way.

RESPONSE: THANKS HAVE REMOVED THE INSIGNIFICANT FINDINGS AND DWELLED ON WHAT WAS SIGNIFICANT TO THIS STUDY.

3. No need to write sub themes like “Factors affecting ART adherence in children…” in discussion. Please discuss the factors without subheadings.

REPSONSE : NOTED THANKS

**Conclusion:**

1. Conclude based on your objective

RESPONSE : THANKYOU, THE FEEDBACK IS NOTED.

**Recommendation:**

2. Recommend based on your result

RESPONSE: THANKYOU, THE FEEDBACK IS NOTED.

**Summary status:** Major revision

RESPONSE: THANKYOU FOR ALL YR COMMENTS AND CONSTRUCTIVE FEEDBACK. I APRRECIATE THE TIME YOU TOOK TO READ THROUGH AND ADVISE. HAVE REVISED MOST OF THE RECOMMENDED CHANGES, AND GIVEN AN EXPLANATION FOR THE REST.

**REVIEWER 2**

First, I would like to congratulate the authors for exploring such an important topic. Magnitude and Attributes Of Anti-Retroviral Therapy Adherence Among Children (1-14 Years) Attending HIV Care And Treatment Clinics In Dar Es Salaam, Tanzania. However I have some comments and questions for them, these are found below

Abstract:

Introduction:

Line 1: In Epidemiological disease level classification HIV is a Pandemic disease; use this term instead of Epidemic. In addition at the end of the introduction please add justification/the need to conduct this study in a line or two.

RESPONSES: CHANGED THE WORD TO PANDEMIC. ADDED A JUSTIFICATION LINE FOR THE STUDY.

Objectives: Correct the English (Among HIV positive children). Objective must be SMART which includes the “T” which stands for time bound. Therefore specify the time of the study.

RESPONSES: CORRECTED THE ENGLISH, SPECIFIED THE TIME

Methods: The first statement is not even a full sentence. The method presented is too shallow. The sample size, the method of data analysis, level of significance, the measurement of association, the model fitness assessment, the software used all are lacking.

RESPONSES: THANKYOU FOR YR CONTRIBUTION; COMPLETED THE SENTENCE. ADDED ALL THE NECESSARY MISSING DETAILS.

Result: The magnitude of good adherence reported (60%) is a point estimate, you should provide the confidence interval. Take the type of analysis used in the method section, use the term multivariable logistic regression instead of multivariate (Multivariate is when you have multiple outcome variables). Age is better reported as a range instead of <25 for children of very young (underage) mothers may not have the same association. Belief in ART effectiveness is not clear (Negative or Positive?? And how did you assess it??). After all the associated factors were written the sentence was concluded as “”… were less likely to be associated poor adherence.” Yet all the significant variables are not negatively associated with the outcome variable. Besides, though, the authors reported the magnitude of good adherence, they reported associated factors for poor adherence, you need to be consistent.

RESPONSES: WE CANNOT DO PREVALENCE AS WE DONOT HAVE THE ENTIRE DENOMINATOR OR TOTAL NUMBER OF CHILDREN ATTENDING THE FACILITY OVER TIME EG. PER YEAR. WE ONLY COUNTED THOSE WHO CAME DURING THE SAID DATES AND CALCULATED A PROPORTION OUT OF THAT FOR GOOD VERSUS BAD ADHERENCE

Conclusion and recommendations: The authors should re-write this section. Most of it is wrong and the recommendations given are not based on the findings.

RESPONSE: THANKYOU FOR YOUR COMMENT. HAVE CONCLUDED AND RECOMMENDED MY STUDY AS I VISIONED IT TO BE AND TO THE BEST OF MY ABILITY.

**Methods and Materials**

Study design and area: In the first line authors mentioned “This was hospital based descriptive cross-sectional study” which is totally wrong. You have assessed factors that affecting adherence therefore it is not mere description. (Use hospital based cross-sectional study)

RESPONSE: CHANGED AS ADVISED

**Participants and eligibility criteria**

The minimum sample size determination must be clearly presented. There is no such a thing as “quota sampling calculation” Quota sampling is a sampling technique (How you distribute the sample size you determined across quotas).

How was the three study places identified?? Why none probability sampling???

How can you take consent form under 14 years old children?? (This must be clearly described in the Ethical consideration)

RESPONSES: MY APOLOGIES THAT WAS A TYPO, I MEANT QUOTA SAMPLING WAS DONE. HAVE CLEARLY SHOWN HOW THE SAMPLE SIZE WAS DERIVED AND HOW WERE THEY DISTRIBUTED AMONGST THE 3 SITES.

THE THREE SITES WERE MORE ACCESSIBLE AND REPRESENTED THREE DIFFERENT LEVELS OF HEALTH FACILITIES IN ORDER TO MAKE THE STUDY REPRESENTABLE IN THE CAPACITY WE HAD.

INFORMED CONSENT WAS SOUGHT FROM THE CAREGIVER WHO BROUGHT THE CHILD TO THE CTC CLINIC. A WRITTEN INFORMED ASSENT WAS SOUGHT FROM CHILDREN WHO WERE CAPABLE OF UNDERSTANDING. HAVE CLARIFIED IT IN THE ETHICAL CONSIDERATION SECTION.

**Variables and measurements**

Clearly put the outcome variable and list of independent variables considered.

RESPONSES: HAVE CLEARLY PUT IT DOWN.

**Statistical analysis**

What method of goodness of fit assessment did you use?? What was its value??

Generally the method part lacks the following: Study period, Sample size determination, Sampling Procedures, Data collection procedure and quality assurance.

RESPONSE: HAVE ADDED THE DETAILS IN THE METHODOLOGY

**Results**

The first paragraph must be part of the method section. You should focus on what you have found and not how you did it in the result section.

The order of the result presentation is full of flaws. First report the characteristics of study participants be it socio-demographic characteristics or co-morbidities. Then go to the descriptive findings of your study which the level/magnitude of adherence, finally you can present the factors associated. The level/magnitude of adherence also needs to be reported using confidence intervals.

RESPONSE: HAVE REMOVED THE FIRST PARAGRAPH AND ARRANGED THE ORDER AS ADVISED.

**Factors associated with ART adherence**

Why are the authors reporting the associated factors as socio-demographic and participant related and the like all variables must be adjusted in the multivariable analysis and be reported as one multivariable table.

RESPONSE: TABLE 2-5 SHOWS THE BIVARIABLE ANALYSIS OF THE FACTORS WE ARE LOOKING INTO CATEGORISED AS SOCIOECONIMIC FACTORS, PARTICIPANT FACTORS, DRUG RELATED FACTORS AND HEALTH SYSTEM RELATED FACTORS. ONLY THOSE WHICH WERE FOUND SIGNIFICANT WERE FURTHER ANALYSED FOR MULTIVARIABLE ANALYSIS AND PUT IN ONE SINGLE TABLE. IT MAY BE DIFFICULT TO PUT ALL THE TABLES IN BIVARIABLE ANALYSIS IN 1 SINGLE TABLE AS WE HAVE CATEGORISED IT. HOWEVER THE MULTIVARIABLE TABLE IS ONE IE: TABLE 6
