# Supplementary material for: Magnitude and associated factors of anti-retroviral therapy adherence among children attending HIV care and treatment clinics in Dar es Salaam, Tanzania
Source: PLoS One. 2022 Sep 30;17(9):e0275420. doi: 10.1371/journal.pone.0275420 (PMC9524636; doi:10.1371/journal.pone.0275420)
Supplement: Attachment — Submitted filename: response to reviewers dec 2021.docx [file pone.0275420.s007.docx]

Response to reviewers

I am thankful to the entire team for their comments and contributions to this manuscript.

These are the responses to the questions posed by Adhanom Gebreegziabher Baraki.

1) I have sent the work to a native English speaker to proofread. I hope it is satisfactory as it stands.

2/3) I have added the confidence interval for adherence and changed the age range 17-25 in the abstract and throughout the manuscript.

4) Pertaining to the direction to interpretation of the negatively associated odds ratio, we have changed the reference variable and made it a positive odds ratio with a more harmonized interpretation. Thus made a change to those 2 variables ie: belief in ART effectiveness and clinical stage variables.

From my reading, odds ratio may be interpreted as odds of having an event occurring was…….. times more or less likely in the exposed group than in the unexposed.

5) Regarding the sampling procedure I have understood what you meant, I have rectified it to Proportion to size sampling rather than quota sampling and explained the technique used in the manuscript.

6) I have indicated the dependent and independent variables considered in this study as requested.

7) In regards to Goodness of fit, I had specified in the results section the Hosmer-Lemshow goodness of fit was used. However I have rephrased it again as requested.

8) Regarding the sample size, I understand that participants are not supposed to be included because they wanted to participate. I apologize for the over enrollment that took place as we were two interviewers collecting simultaneously at different sites, So that led to over recruitment due to lack of communication. Once we collected the data, we felt it unwise to discard the data. Thankyou for pointing that out.

9) The final multivariate table has been re-visited and all the variables required in cooperated.
