# Supplementary material for: Magnitude and associated factors of anti-retroviral therapy adherence among children attending HIV care and treatment clinics in Dar es Salaam, Tanzania
Source: PLoS One. 2022 Sep 30;17(9):e0275420. doi: 10.1371/journal.pone.0275420 (PMC9524636; doi:10.1371/journal.pone.0275420)
Supplement: Attachment — Submitted filename: Review Report.docx [file pone.0275420.s008.docx]

TITLE: **MAGNITUDE AND ASSOCIATED FACTORS OF ANTIRETROVIRAL THERAPY ADHERENCE AMONG CHILDREN ATTENDING HIV CARE AND TREATMENT CLINICS IN DAR ES SALAAM, TANZANIA**

**REVIEW REPORT**

1. **Summary of the Research**

The paper presents a cross-sectional clinic-based study to assess the magnitude of, and factors associated with adherence to antiretroviral therapy (ART) among children aged 1-14 years living with HIV and accessing HIV care from 3 health facilities in Dar es Salaam, Tanzania in 2018. Three measures (4-day self-report, one-month self-recall, and missed clinic appointments) were used to assess adherence. Logistic regression statistics were used to obtain socio-demographic and HIV-related factors that were significantly associated with adherence. Results showed that 60% of participants had good (≥ 95%) adherence to ART, and that poor adherence was significantly associated with caregivers aged 17-25 years, intercurrent illness in the child, disbelief in the effectiveness of ART, and advanced disease stage in the child.

The paper is significant in that it highlights the low ART adherence in children responsible for the poorer HIV treatment outcomes in children in Africa as well as some of the factors associated with poor adherence. This will help to streamline strategies to improve ART adherence in children living with HIV.

2. **Examples and Evidence**

There are no major issues identified in the paper.

However, some minor issues identified are:

1. Line 4 of the 2nd paragraph of the introduction says 1.7 million children (1-14 years) were living with HIV and 1.9 million of them were in sub-Saharan Africa. I think this is a typo as the number is about 1.09 million (64% of the 1.7 million).

2. In statistical analysis, it is mentioned that measures of central tendency were used to summarize discrete data; the only measure of central tendency that can be applied to discrete data is mode and this should be stated if it was used.

3. In the results section: for age groups, 25 years appears in 2 strata: 17-25 years and 25-34 years. This should be appropriately revised; either 17 -<25 years and 25-35 years, OR 17-25 years and >25 - 34 years.
