# Supplementary material for: Magnitude and associated factors of anti-retroviral therapy adherence among children attending HIV care and treatment clinics in Dar es Salaam, Tanzania
Source: PLoS One. 2022 Sep 30;17(9):e0275420. doi: 10.1371/journal.pone.0275420 (PMC9524636; doi:10.1371/journal.pone.0275420)
Supplement: Attachment — Submitted filename: RESPONSE TO REVEIWERS.docx [file pone.0275420.s009.docx]

**RESPONSES TO REVIEWERS**

Dear editor and reviewer, Thankyou for taking up this review process again. As you may have already known, this work had gone through an extensive review process over the past one and half year, I’m not sure which version of the work you have finally reviewed as some of the comments had already been addressed and edited in the last version. However, I have tried to answer your valid comments in the best of my capacity. Im proud to claim that my work has been validated by 6 reveiwers and 2 editors!!!

Reviewer #2’comments

Comment 1: Better to put AOR which are significant (i.e., p<=0.05) instead of OR as final results in the results section for factors-

Response : have addressed the suggested valid comments in the manuscript.

Comment 2: In the conclusion section, factors contributing to low adherence from Table 6 must be mentioned (like age of care giver, child inter-current illness, believes on effectiveness of ART, and clinical stage).

Response: it was already mentioned in the conclusion of the discussion section but was missing in the abstract, I have now included it.

Comment 3: Usually, sample sizes are denoted by n and population by N. So, better to denote your sample size by n.

Resposnse: Have denoted the sample sizes as n

Reviewer #4 comments
comment 1: Study Design and study area:
Reference was made to National guidelines. It is useful to state the country of origin of the guidelines. Is this the Tanzanian National guidelines? Please clarify.

Response: Yes, I meant the Tanzanian National guidelines for HIV/AIDS. I have clarified it in the document.

comment 2: Methodology is silent on the specific factors affecting adherence only to surface in the results. Unless this is the journal preference, mention should be made in methodology of the specific factors affecting ART adherence.

Response: This comment was already addressed in the revised latest version which I assume you may not have seen, I have highlighted in the document again.

Comment 3: The description of the quota sampling technique was inadequate. This requires more detail to understand the populations of the patients at the CTC sites and how representative the sampled research participants are of the CTC sites. Given the drawbacks of Quota Sampling methodology – Non-probability sampling, researcher’s bias in selection of study participants and limitations with generalization of study findings, a lot more detail would be required. Was the sampling controlled or not controlled for instance.

Response: this also was addressed in the previous review and after discussion, it was concluded that it was not a quota sampling (a misnomer) but rather a proportion to size sampling technique based on the number of patients enrolled in the specific site in order to obtain a representative yet unbiased sample based on the site of recruitment, where participants where then consecutively recruited till the number was reached from each site and eventually the required sample size. I added a better clarification in the manuscript.

Comment 4: Results
Socio-demographic factors affecting adherence: a. Child age grouping of 1-9 years and 10-14 years lumps children with different needs together. The Under-five children age group is entirely dependent on the caregiver for medication administration as opposed to the older age groups. School-age children attending day school would have different challenges when compared with children in boarding school. Combining all these children together into just two groups with different needs may hide information that is inimical to adherence. Consider age groups of U5, 6-10, 11-14.
b. Similarly, parental grouping could be further revised to Single parents, both parents, grandparents and other caregivers. The challenges for each group differ from the other and may impact differently on adherence.
c. Care giver marital status also appears to be lumped together. Married, cohabiting, single, Divorced, widowed caregivers could be assessed separately. Married and cohabiting on one hand and single divorced and widowed caregivers on the other hand may not have the same challenges. It is possible that evaluating each group may be more informative that combining them.

Response: Thank you for this comment, in regards to the child age group we had considered this among many other sub-categorization methods, however during our data collection process we found that under 9 years in our setting were totally dependent on the caregiver for their overall needs and HIV care specifically such that even drugs were being administered solely by the caregiver, even boarding school options were not there for this category. Thus, we deemed it wise to categorize them as we have done due to the similarities. Generally, in this setting children below 10 years are still considered to be totally dependent on their caregivers.

In regards to parental status, we thought it would be wise to look into whether a parental presence would affect the outcome of interest, whether one or both or none as we know a parent would strive hard for their own child as compared to others even if it is a single parent. In our setting, it is quite common to have different partners, married or not at different times, so having another partner instead of the child’s own another parent would not change the outcome and it might be culturally not acceptable to ask if you are still living with the father/mother of the index child, especially if they had no short-term or long-term relationship which is very customary here. Similarly, we opted to group the marital status in a culturally unoffending way as married/cohabiting as we know atleast they have a partner support versus the single/divorced/widowed with no partner support. Cohabiting is culturally accepted here and it may be hostile to ask them whether they’re living together out of wedlock or they have actually married each other.

Conclusion:
Comment 5. Pill burden
Specify the type of pill burden, high or low.

Response: have specified it as high, thankyou.

Recommendation:
comment 6. Adherence counseling: The authors’ results show that there is no association between lack of adherence counseling and poor ART adherence and yet recommended the necessity of counseling. This needs clarification.

Response: ART adherence was still found to be sub-optimal at around 60%, the most important strategy to improve ART adherence is to strengthen counselling, which in our study is reported to be done, however, may still need to be reinforced in the high risk groups like younger aged caregivers, sick children as these children are still dependant of their caregivers. Younger aged caregivers being less mature than their counterparts may need more support and education. Sick children and those with advanced HIV stages may need to be admitted and have closer follow-ups with their clinicians to improve their condition and improve adherence. Health talks during clinic visits and on social media platforms may improve belief and alleviate misconceptions in the ARTs. There’s no single way to predict patients’ adherence or nonadherence status and it has been seen that adherence is always better earlier in the disease in patients who are more motivate and less fatigued, it is ongoing adherence measurements and early identification of faltering adherence and continuing counselling that may be able to bring it back up. Thus these may not be separable and is vital for ART adherence success.

Reviewer #5: 
comment 1: Line 4 of the 2nd paragraph of the introduction says 1.7 million children (1-14 years) were living with HIV and 1.9 million of them were in sub-Saharan Africa. I think this is a typo as the number is about 1.09 million (64% of the 1.7 million).

Response: Amends has been made, thanks.

comment 2. In statistical analysis, it is mentioned that measures of central tendency were used to summarize discrete data; the only measure of central tendency that can be applied to discrete data is mode and this should be stated if it was used.

Response: Age in this study was taken in years not considering the months, and median was applied to it to find the median age of children as our age outliers. Mode was used by default for all categorical data.

comment 3. In the results section: for age groups, 25 years appears in 2 strata: 17-25 years and 25-34 years. This should be appropriately revised; either 17 -<25 years and 25-35 years, OR 17-25 years and >25 - 34 years.

This has been addressed and changed accordingly, thank you.
