# Supplementary material for: Magnitude and associated factors of anti-retroviral therapy adherence among children attending HIV care and treatment clinics in Dar es Salaam, Tanzania
Source: PLoS One. 2022 Sep 30;17(9):e0275420. doi: 10.1371/journal.pone.0275420 (PMC9524636; doi:10.1371/journal.pone.0275420)
Supplement: Attachment — Submitted filename: Reviewer comment for PONE-D-20-25583.docx [file pone.0275420.s004.docx]

First, I would like to congratulate the authors for exploring such an important topic. Magnitude and Attributes Of Anti-Retroviral Therapy Adherence Among Children (1-14 Years) Attending HIV Care And Treatment Clinics In Dar Es Salaam, Tanzania. However I have some comments and questions for them, these are found below

Abstract:

Introduction:

Line 1: In Epidemiological disease level classification HIV is a Pandemic disease; use this term instead of Epidemic. In addition at the end of the introduction please add justification/the need to conduct this study in a line or two.

Objectives: Correct the English (Among HIV positive children). Objective must be SMART which includes the “T” which stands for time bound. Therefore specify the time of the study.

Methods: The first statement is not even a full sentence. The method presented is too shallow. The sample size, the method of data analysis, level of significance, the measurement of association, the model fitness assessment, the software used all are lacking.

Result: The magnitude of good adherence reported (60%) is a point estimate, you should provide the confidence interval. Take the type of analysis used in the method section, use the term multivariable logistic regression instead of multivariate (Multivariate is when you have multiple outcome variables). Age is better reported as a range instead of <25 for children of very young (underage) mothers may not have the same association. Belief in ART effectiveness is not clear (Negative or Positive?? And how did you assess it??). After all the associated factors were written the sentence was concluded as “”… were less likely to be associated poor adherence.” Yet all the significant variables are not negatively associated with the outcome variable. Besides, though, the authors reported the magnitude of good adherence, they reported associated factors for poor adherence, you need to be consistent.

Conclusion and recommendations: The authors should re-write this section. Most of it is wrong and the recommendations given are not based on the findings.

**Methods and Materials**

Study design and area: In the first line authors mentioned “This was hospital based descriptive cross-sectional study” which is totally wrong. You have assessed factors that affecting adherence therefore it is not mere description. (Use hospital based cross-sectional study)

**Participants and eligibility criteria**

The minimum sample size determination must be clearly presented. There is no such a thing as “quota sampling calculation” Quota sampling is a sampling technique (How you distribute the sample size you determined across quotas).

How was the three study places identified?? Why none probability sampling???

How can you take consent form under 14 years old children?? (This must be clearly described in the Ethical consideration)

**Variables and measurements**

Clearly put the outcome variable and list of independent variables considered.

**Statistical analysis**

What method of goodness of fit assessment did you use?? What was its value??

Generally the method part lacks the following: Study period, Sample size determination, Sampling Procedures, Data collection procedure and quality assurance.

**Results**

The first paragraph must be part of the method section. You should focus on what you have found and not how you did it in the result section.

The order of the result presentation is full of flaws. First report the characteristics of study participants be it socio-demographic characteristics or co-morbidities. Then go to the descriptive findings of your study which the level/magnitude of adherence, finally you can present the factors associated. The level/magnitude of adherence also needs to be reported using confidence intervals.

**Factors associated with ART adherence**

Why are the authors reporting the associated factors as socio-demographic and participant related and the like all variables must be adjusted in the multivariable analysis and be reported as one multivariable table.
